# Low-Temperature Sintering of l-Alanine-Functionalized Metallic Copper Particles Affording Conductive Films with Excellent Oxidative Stability

**DOI:** 10.1021/acsaelm.2c00275

**Published:** 2022-05-03

**Authors:** H. Jessica Pereira, C. Elizabeth Killalea, David B. Amabilino

**Affiliations:** The GSK Carbon Neutral Laboratories for Sustainable Chemistry, School of Chemistry, University of Nottingham, Nottingham NG7 2TU, United Kingdom

**Keywords:** metallic copper, green chemistry, aqueous synthesis, low-temperature sintering, conductive films, passivation

## Abstract

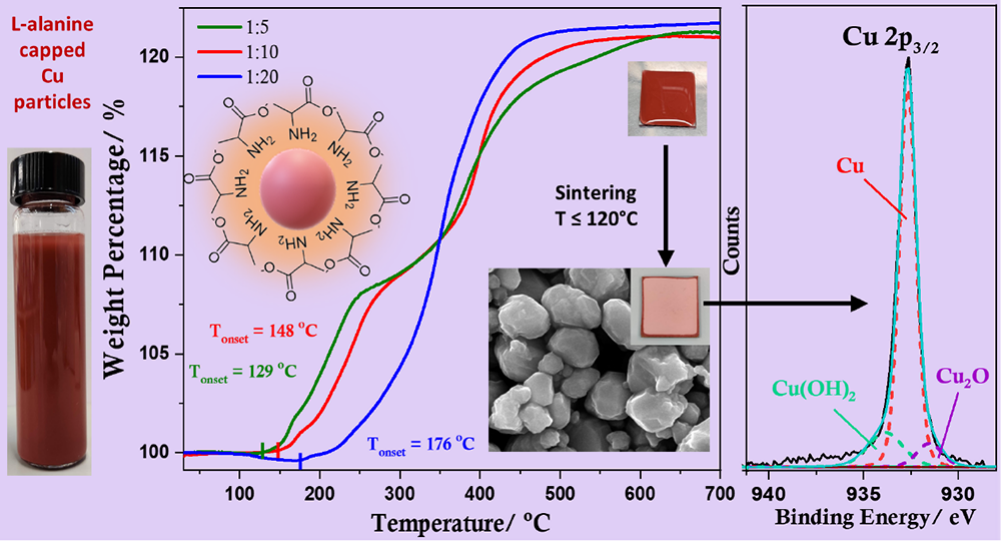

Here, the alpha amino acid l-alanine is employed as both a capping and stabilizing agent in the aqueous synthesis of submicron-sized metallic copper particles under ambient atmospheric conditions. The reduction of the copper(II) precursor is achieved using l-ascorbic acid (vitamin C) as the reducing agent. The nature of the complex formed between l-alanine and the copper(II) precursor, pH of the medium, temperature, and the relative proportion of capping agent are found to play a significant role in determining the size, shape, and oxidative stability of the resulting particles. The adsorbed l-alanine is shown to act as a barrier imparting excellent thermal stability to capped copper particles, delaying the onset of temperature-induced aerial oxidation. The stability of the particles is complemented by highly favorable sintering conditions, rendering the formation of conductive copper films at significantly lower temperatures (*T* ≤ 120 °C) compared to alternative preparation methods. The resulting copper films are well-passivated by residual surface l-alanine molecules, promoting long-term stability without hindering the surface chemistry of the copper film as evidenced by the catalytic activity. Contrary to the popular belief that ligands with long carbon chains are best for providing stability, these findings demonstrate that very small ligands can provide highly effective stability to copper without significantly deteriorating its functionality while facilitating low-temperature sintering, which is a key requirement for emerging flexible electronic applications.

## Introduction

Metallic copper particles with nano (<100 nm) and submicron (<1 μm) dimensions are emerging as an attractive alternative to silver for diverse applications.^1^ Copper offers significant potential advantages over silver, exhibiting similar conductivity (Cu: 5.96 × 10^7^ S/m *vs* Ag: 6.30 × 10^7^ S/m), at around a hundredth of the cost of silver, because of its relative abundance in the Earth’s crust (Cu: 60 mg/kg *vs* Ag: 0.075 mg/kg).^2^ However, nano or submicron copper particles have been somewhat limited in their adoption in technologies, because of their propensity to undergo rapid oxidation^3^ compared to their bulk counterpart owing to the inevitable increase in surface to volume ratio at these dimensions and the higher reactivity compared to other coinage metal particles. This process results in the rapid formation of surface oxides (Cu_2_O and CuO (*via* Cu(OH)_2_ in the presence of moisture and oxygen),^4^ which affect the interfacial properties of the material, particularly, its electrical conductivity. Although both oxides are *p*-type semiconductors, their electrical conductivity is significantly lower (0.1–0.01 S/m for Cu_2_O and 10^2^–10^4^ S/m for CuO)^5^ compared to that of the pristine metal.

Several approaches including (i) adopting a core–shell structure with a more atmospherically stable metal such as gold, silver, or nickel forming the outer shell;^3,6,7^ (ii) alloying of copper;^8^ (iii) use of metal oxides or aluminum-doped zinc oxide as the shell material in core–shell structures;^9,10^ (iv) employing various forms of graphene as a barrier layer;^3,11^ and (v) use of organic ligands as capping agents^3,12^ have been explored to achieve improved stability. Although these approaches do limit the oxidation of copper, the properties of the metal are significantly impacted by the overall properties of the hybrid structure.

Organic ligands are an attractive choice as stabilizing agents; however, the typical approach is to use ligands featuring long carbon chains, as that will effectively form a hydrophobic barrier around the particle improving stability. Most common ligand choices include hexadecyltrimethylammonium bromide (CTAB),^3,13,14^ alkyl amines (hexyl, octyl, hexadecyl),^15,16^ thiol-terminated chains,^12^ carboxylic acid ligands,^17,18^ and polymers such as polyvinylpyrrolidone.^19^ Although these relatively larger ligands confer significant stability to copper particles, the long carbon chain leads to increased distance between neighboring particles. This means that the capping material has to be nearly fully decomposed before contact can be established between adjacent particles when fabricating conductive films, thus requiring high sintering temperatures that approach the decomposition temperature of the capping ligand or polymer. In the case of thermal sintering, depending on the particle dimensions, the atmosphere (N_2_, H_2_, or vacuum), capping ligand, and solvents used, the exact temperature required for conductive film formation can vary significantly but typically lies between 150 and 350 °C.^1,20,21^ If the compact monolayers of long saturated ligands are not decomposed, they can act as insulators, disrupting electron transport, resulting in relatively poor electrical conductivity compared with the native metal.^12,22^ Conversely, their complete removal exposes the copper particles, retarding stability significantly. Some relatively smaller capping agents such as 1-amino-2-propanol^21^ and nitrilotriacetic acid disodium salt^20^ have been used to achieve lower temperature sintering compared to other reports; however, some of these capping ligands and other chemicals used in the synthesis can be associated with hazards imposing a threat to safety (Table S1).

Apart from a capping/stabilizing agent, a strong reducing agent is required to convert the copper salt to the metal. Hydrazine is the most commonly used reducing agent for this purpose.^18,23^ Another popular choice is oleylamine, which serves both as a solvent and stabilizing agent.^12,16^ Several other chemicals including monoethylene glycol, isophorone, and diethylene glycol monobutyl ether^24,25^ are also used in commercial copper ink formulations as solvents/additives. However, as summarized in Table S1, most have significant drawbacks in terms of their toxicity and sustainability. Their use also increases the overall cost of production because of excess toxic waste generation and waste treatment, which is essential for safe disposal. Recently, a handful of aqueous or biogenic synthetic approaches using benign reducing agents supported by appropriate stabilizing agents and surfactants have been reported,^26−29^ where all chemicals and solvents used can be strictly categorized as sustainable or “green” (Table S1). However, although these reports^27−31^ focus on the influence of reaction conditions and reagent ratios on size and shape of the particles, technologically important parameters have not been studied extensively.

To this end, we report the sustainable aqueous synthesis of submicron-sized metallic copper particles with important technological properties including high thermal stability, excellent oxidative stability (using surface sensitive X-ray photoelectron spectroscopy (XPS)), low-temperature sintering capability, electrical conductivity, and catalytic activity. This work uses l-ascorbic acid as the reducing agent and l-alanine as the particle capping agent, both of which are highly benign. l-Ascorbic acid is a naturally occurring vitamin found in many fruits with very good reducing ability and antioxidant effects. The latter makes ascorbic acid very attractive in the synthesis of copper particles and preparation of films, as it can retard oxidation owing to its oxygen scavenging abilities.^32^l-Alanine is a non-essential alpha amino acid that is used in the biosynthesis of proteins. It is used as a component in infusion solutions, a precursor in the synthesis of pharmaceutical products, and in the food industry.^33^

Amino acids are an interesting class of ligands as they possess amine as well as carboxylic acid terminal groups, which are important as structure-directing agents, determining the size and shape of the particles formed. Furthermore, the presence of additional functional groups in some amino acids could also be beneficial. Specifically, l-cysteine^29,34^ has been studied extensively owing to the presence of an additional thiol functionalization, which is known to interact strongly with metallic copper particles.^12^ However, to the best of our knowledge, l-alanine has been used once previously in the synthesis of copper nanoparticles by Deng *et al.*(18) and in the synthesis of short nanowires by Yu *et al.*(35) In both reports, l-alanine has been used in conjunction with hydrazine hydrate as the reducing agent. Moreover, although nanoparticles/short nanowires were synthesized, the effect of proportion of l-alanine and reaction conditions on size and shape of resulting particles, low-temperature thermal sintering to form conductive films, long-term stability, thermal stability, and surface properties including catalytic activity have not been previously reported. Therefore, l-alanine was selected as the capping agent for this study as it is a small amino acid, comprising only three carbon atoms, hence a suitable capping agent for the investigation of low-temperature sintering because diffusion and electron transport through the layer should be favorable.

Thus, our sustainable approach for the synthesis of copper particles is distinct in several aspects: (i) All chemicals and solvents used are truly benign and strictly follow the tenets of sustainable and green chemistry (particularly aligned with the concepts of using less hazardous synthesis protocols, safer solvents and auxiliaries, and inherently safer chemistry for accident prevention and minimizing waste generation^36^); (ii) low-temperature synthesis under ambient atmospheric conditions; (iii) stable aqueous synthesis resulting in significantly low surface oxides as evidenced by XPS studies; and (iv) low-temperature thermal sintering – in the temperature range of 80–120 °C under vacuum – to produce electrically conducting copper films with enhanced long-term stability. Furthermore, owing to the low-temperature sintering capability, these l-alanine-capped copper particles could be potential candidates for emerging copper inks, and as demonstrated, they are well-suited for flexible substrates, which are unable to withstand high temperatures, typically ≥150 °C.

## Experimental Section

### Materials

Copper(II) chloride dihydrate (CuCl_2_·2H_2_O, 99%, Fisher Scientific), l-alanine (≥99%, Sigma-Aldrich), l-ascorbic acid (99+%, Alfa Aesar), sodium hydroxide (99+%, Alfa Aesar), 4-nitrophenol (98%, Acros Organics), sodium borohydride (NaBH_4_, 99%, Acros Organics), absolute ethanol (99%, Fisher chemical), IPA (99.5%, Fisher chemical), acetone, microscope slides (Biosigma), Hellmanex III (Ossila), and disposable polypropylene centrifuge tubes (50 mL, Fisher Scientific) were used.

### Synthesis of Copper Particles

Aqueous solutions (80 mL) containing CuCl_2_·2H_2_O (10 mM) and various amounts of l-alanine (50, 100, 150, and 200 mM) were prepared and heated to 75 °C in air. Upon reaching this temperature, NaOH (1.25 M) was added dropwise until the pH of the solution reached 10. l-Ascorbic acid (1.3 M, 15 mL) was then added into the reaction mixture *via* a syringe pump at a rate of 0.25 mL min^–1^. The gradual color change from deep blue, green, yellow, orange to red is observed as l-ascorbic acid is added. The reaction mixture was maintained at 75 °C and stirred at 450 rpm until the addition of l-ascorbic acid was complete. After an additional 30 min, the reaction mixture was cooled and centrifuged using a Thermo Scientific Heraeus Megafuge 8 centrifuge at 8000 rpm for 15 min to separate the copper particles. The solid was washed with absolute ethanol and dried under vacuum to isolate the copper powder. The dried copper was stored in sealed vials. (All experiments were performed under ambient conditions, unless otherwise stated.)

### Formation of Copper Films

Glass substrate slides were cut (25 × 25 mm) from microscope slides and cleaned by sonicating consecutively in a diluted solution of surfactant (Hellmanex III), deionized water, acetone, and IPA for 20 min each followed by drying with a stream of nitrogen. Dried copper particles (40 mg/mL) were dispersed in a 1:1 solution of absolute ethanol and aqueous l-ascorbic acid (15 mM) and drop-cast (350 μL) on the cleaned glass substrates. The substrates were then thermally sintered (80–120 °C for 45/120 min) under vacuum using a PELCO Mini Hot Vac vacuum desiccator. After the samples reached room temperature, a Keithley 2401 source measurement unit was used to compute the sheet resistance using the van der Pauw method. The thickness of the deposited film was found to be 7.1 ± 1.3 μm using a KLA-Tencor Alpha-Step D120 Stylus profilometer.

### Characterization

The morphology of the particles was studied using a JEOL 2100F transmission electron microscope (TEM) operating at 200 kV equipped with a Gatan Orius CCD camera and JEOL scanning transmission electron microscopy (STEM) detectors. Image J software was used to analyze the TEM images and compute particle size distributions. Energy-dispersive X-ray spectroscopy (EDS) acquisition was by an Oxford Instruments 80 mm X-Max detector and INCA software and processed with Oxford Instruments Aztec software. Selected-area electron diffraction (SAED) patterns were also acquired with the Gatan Orius CCD camera. Calibration was done using an evaporated aluminum film as a reference (from Agar Scientific). UV–visible absorption spectra were obtained using an Agilent Cary UV–Vis–NIR spectrophotometer. For TEM and UV–Vis analysis, copper particles were dispersed in a 1:1 solution of absolute ethanol/IPA and aqueous l-ascorbic acid (15 mM). Zeta potential measurements were performed using a Malvern Zetasizer (copper particles were dispersed in absolute ethanol) together with DTS1070 disposable folded capillary cells purchased from Malvern Instruments. Thermogravimetric analysis (TGA) was performed using a TA instruments Discovery TGA instrument. Platinum HT sample pans (957571.901) were used for the analysis. X-ray powder diffraction (XRD) data was collected using a Panalytical material research diffractometer. Loading of samples and data acquisition were performed in air. The XRD peaks were assigned using the Mercury software and the CDS National Chemical Database. XPS was performed using the Kratos AXIS ULTRA with a monochromated Al Kα X-ray source (1486.6 eV) operated at 10 mA emission current and 12 kV anode potential (120 W). Spectra were acquired with the Kratos VISION II software. A charge neutralizer filament was used to prevent surface charging. Hybrid-slot mode was used for measuring a sample area of approximately 300 × 700 μm. The analysis chamber pressure was greater than 5 × 10^–9^ mbar. Three areas per sample were analyzed. A wide scan was performed at low resolution (binding energy range 1400 eV to −5 eV, with pass energy of 80 eV, step of 0.5 eV, sweep time of 20 min). High-resolution spectra at a pass energy of 20 eV, step of 0.1 eV, and sweep time of 10 min each were also acquired for photoelectron peaks from the detected elements, and these were used to model the chemical composition. Sample loading to the XPS was performed in air. The spectra were charge-corrected by setting the C 1s peak to 284.7 eV. Morphologies of the thermally sintered copper films supported on glass substrates were studied using a JEOL 7100 field emission gun scanning electron microscope (FEG-SEM) with an accelerating voltage of 15 kV.

### Catalytic Activity of Sintered Copper Films

An aqueous solution of 4-nitrophenol (10^–5^ M) was prepared followed by addition of excess NaBH_4_ (0.01 g). The above solution (3 mL) was transferred to a cuvette together with a 4 × 4 mm sample of a sintered copper film (thickness of 7.1 ± 1.3 μm) supported on flexible polyethylene terephthalate, and the UV–Vis absorption was recorded at 90 s intervals. As a result of the small size and light weight of the flexible substrate, the copper film moves to the top surface of the solution, resulting in no disruption during the measurement. Upon complete conversion of 4-nitrophenol to 4-aminophenol, the copper film was carefully removed from the cuvette, gently washed with deionized water, and reused in a similar reaction. This sequence was repeated for five consecutive cycles. The reaction rate was calculated assuming pseudo-first-order kinetics with respect to 4-nitrophenol.^37^

## Results and Discussion

**Scheme 1:**
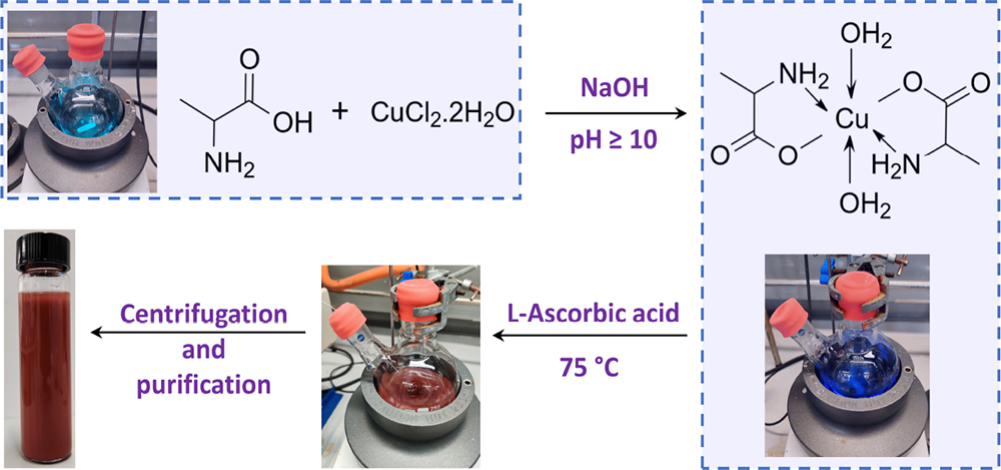
Reaction Scheme Showing the Key Steps in the Synthesis of l-Alanine-Capped Copper Particles

Copper particles were synthesized in an aqueous medium under ambient atmospheric conditions using l-ascorbic acid to chemically reduce Cu(II) to Cu(0). The particles were first capped with l-alanine (Scheme 1), an α-amino acid consisting of a carboxylic group (p*K*_a_ value of 2.34) and an NH_3_^+^ group (p*K*_a_ of 9.87).^2^ Therefore, a pH of ∼10 is essential to ensure that both these groups are deprotonated, hence available for complexing with Cu(II) ions supplied from the precursor. Upon addition of l-alanine to Cu(II), a light blue solution is formed, which turns into an intense deep blue color when the pH is gradually increased to 10, resulting in the formation of a bidentate complex with l-alanine.^38^ Subsequently, upon introducing l-ascorbic acid into the reaction mixture, copper is gradually reduced to Cu(I) and finally to Cu(0).

In order to investigate the effect of l-alanine on the stability of the copper particles, the content of l-alanine was varied so that the Cu(II):l-alanine ratios were 1:5 (Cu-Ala5), 1:10 (Cu-Ala10), or 1:20 (Cu-Ala20). The pH of the medium, concentration of l-ascorbic acid, rate of addition of l-ascorbic acid, temperature of the reaction mixture, and amount of l-alanine influenced the size and shape of the synthesized copper particles (Figure S1). At pH = 8–9, a high rate of introducing the reducing agent, and at lower temperatures (45–65 °C), a mixture of rods as well as particles were observed (Figure S1). This result could be attributed to an alteration in the nature of the complex formed between Cu(II) and l-alanine, as a pH below 10 is insufficient to deprotonate the NH_3_^+^ group, hindering the formation of the bidentate complex, since the nitrogen atom of l-alanine is not available for complexation. Therefore, the nucleation and growth of particles are affected rendering a change in the shape.^39^ At temperatures between 45–65 °C, the rate of growth is such that both rods and particles are formed. Although higher concentrations of ascorbic acid (1.7 and 1.9 M) yielded slightly smaller particles, a mix of particles and rods was observed and therefore was unsuitable for this particular study. Thus, in order to synthesize particles, an l-ascorbic acid solution having a concentration of 1.3 M introduced to the reaction mixture at a rate of 0.25 mL min^–1^ was deemed optimum.

**Figure 1:**
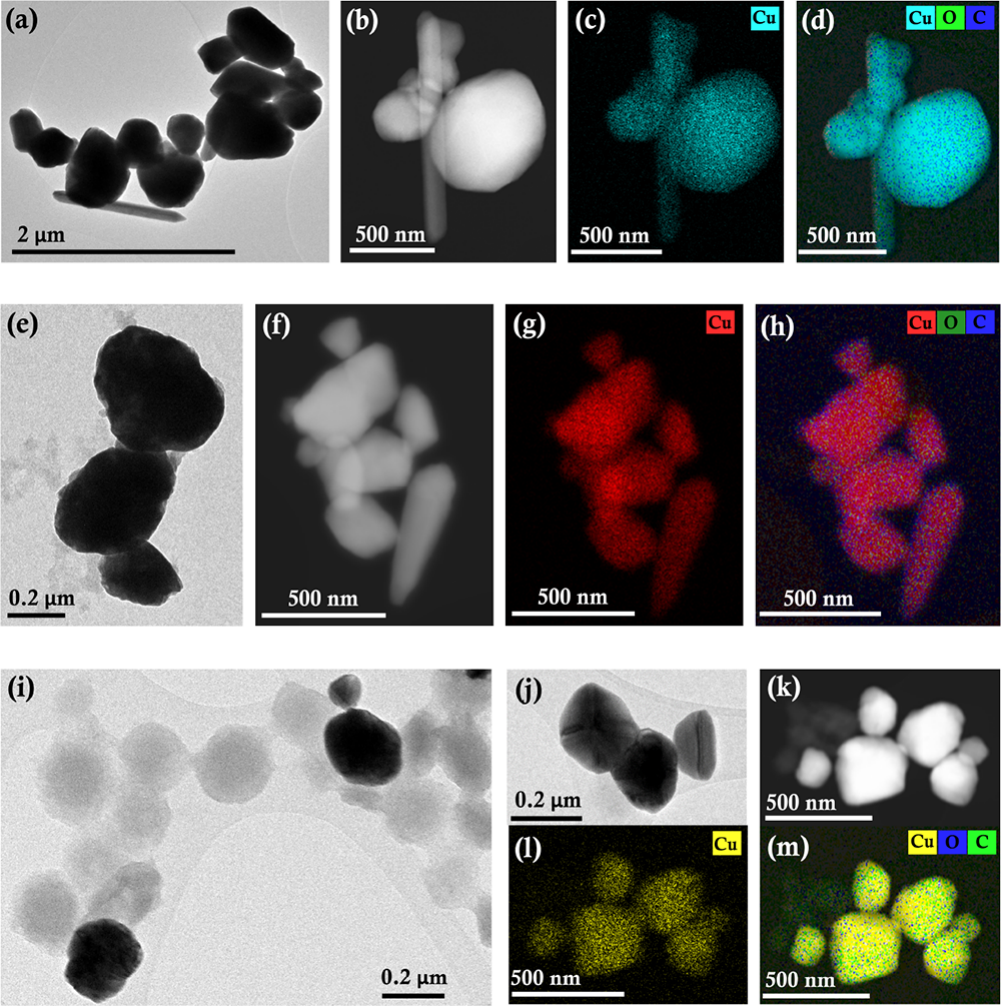
Representative TEM (a,e,i,j); high-angle annular dark-field (HAADF)/annular dark-field (ADF) – STEM images (b,f,k) and STEM -EDS elemental maps of the copper particles corresponding to copper (c,g,l); and overlays (d,h,m); showing the distribution of copper, oxygen, and carbon for copper particles with a Cu(II):l-alanine ratio of 1:5 (a–d); 1:10 (e–h), and 1:20 (i–m).

The R-group in the amino acids has been shown to greatly influence the shape of the copper particles formed.^35^ This work also demonstrates that the same ligand can be employed to synthesize different shapes and sizes of copper particles by varying the aforementioned reaction conditions. It is plausible to suggest that a mix of rods and particles observed at Cu-Ala5 and Cu-Ala10 samples is likely caused by a structuring role of l-alanine, which is influenced by the relative proportions of Cu(II) ions and l-alanine. This type of role is not surprising as amino acids are used as structure-directing agents enabling control of the size and shape of metal nanoparticles.^35^ However, this observation could also be attributed to the complex formation of l-alanine in water,^40^ which could in turn affect the manner in which l-alanine interacts with copper depending on whether they are free l-alanine molecules or l-alanine complexes. For the purpose of this study, the reaction conditions were controlled (see the Experimental Section) in order to favor the formation of particles with a very low proportion of rods. The presence of rods diminishes as the proportion of l-alanine increases, and they are undetectable in the Cu-Ala20 sample (Figure S1 and Figure 1).

**Figure 2:**
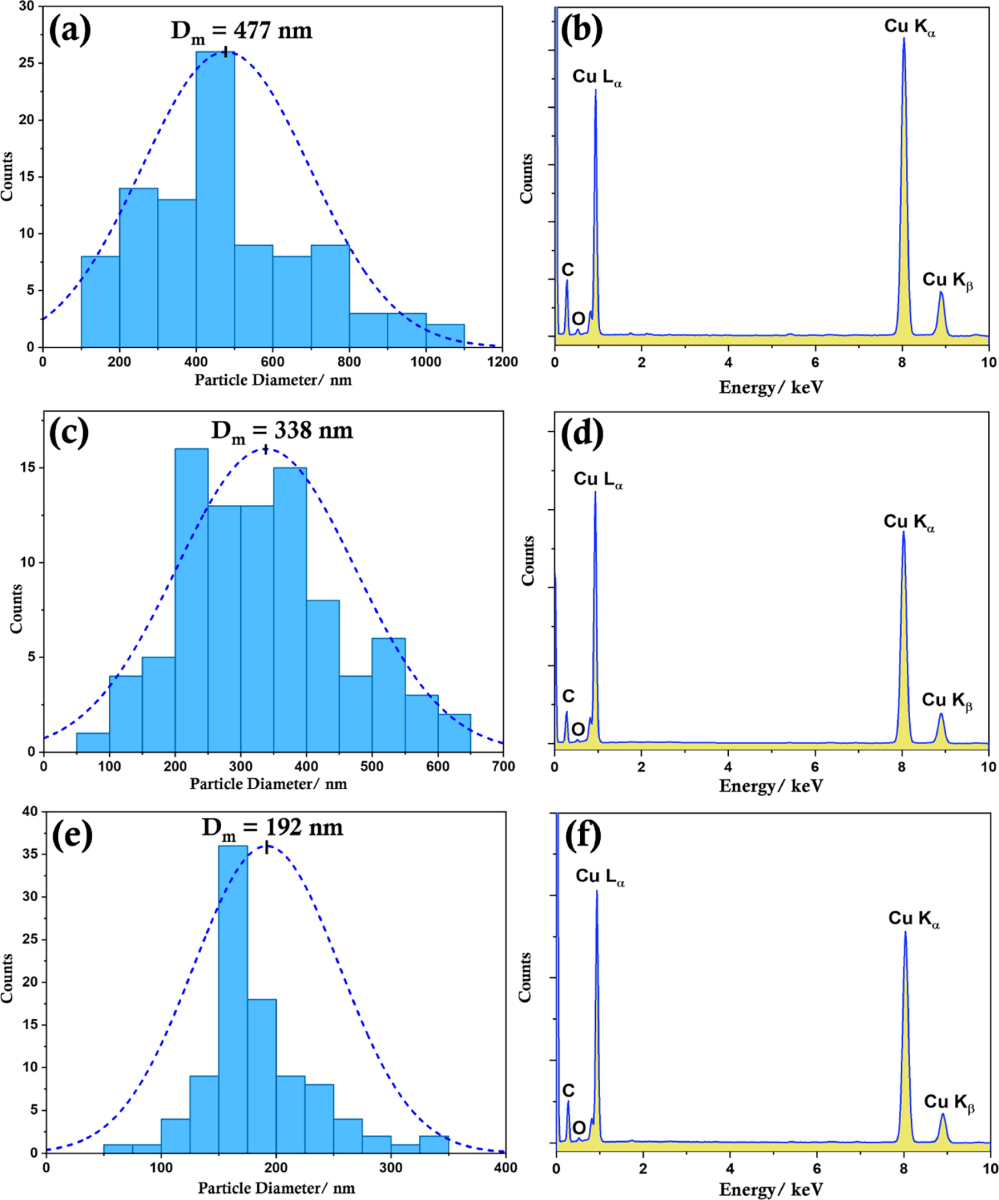
Representative particle size distributions (a,c,e) and EDS analysis of copper particles (b,d,f) corresponding to a Cu(II):l-alanine ratio of 1:5 (a,b); 1:10 (c,d), and 1:20 (e,f). Particle size distribution has been computed considering at least 100 particles for each type.

The TEM images, corresponding size distributions, and EDS data are shown in Figures 1 and 2. It is clear that an increase in the amount of l-alanine decreases the particle size. This diminution is accompanied by a narrowing of the distribution of particle sizes, with Cu-Ala5 showing the broadest distribution and Cu-Ala20 showing a distribution of particle sizes between 50 and 350 nm with a mean of ∼192 nm. The corresponding elemental maps and EDS data confirm that the particles are copper, together with the presence of oxygen and carbon (Figure S2). The existence of nitrogen, however, is not confirmed by EDS measurements, perhaps because this element is present in very low amounts and therefore was undetected by EDS (as the amounts of carbon and oxygen are also very low in intensity compared to copper and nitrogen is expected to be much lower). However, as will be shown later, more surface-sensitive XPS confirms the presence of nitrogen and therefore l-alanine on the surface of the synthesized copper particles.

The *d* spacings calculated from the SAED patterns (Figure S3) collected for the copper particles are in good agreement with the standard ICSD – 43493, indicating the presence of an FCC (space group: *Fm*3̅*m*) crystal structure. The crystal structure is further corroborated by X-ray powder diffraction studies (Figure S4). Notably, no evidence of oxides is observed even though sample preparation was performed in air and each sample was exposed to air for at least 40 min from loading the sample to completion of data acquisition.

**Figure 3:**
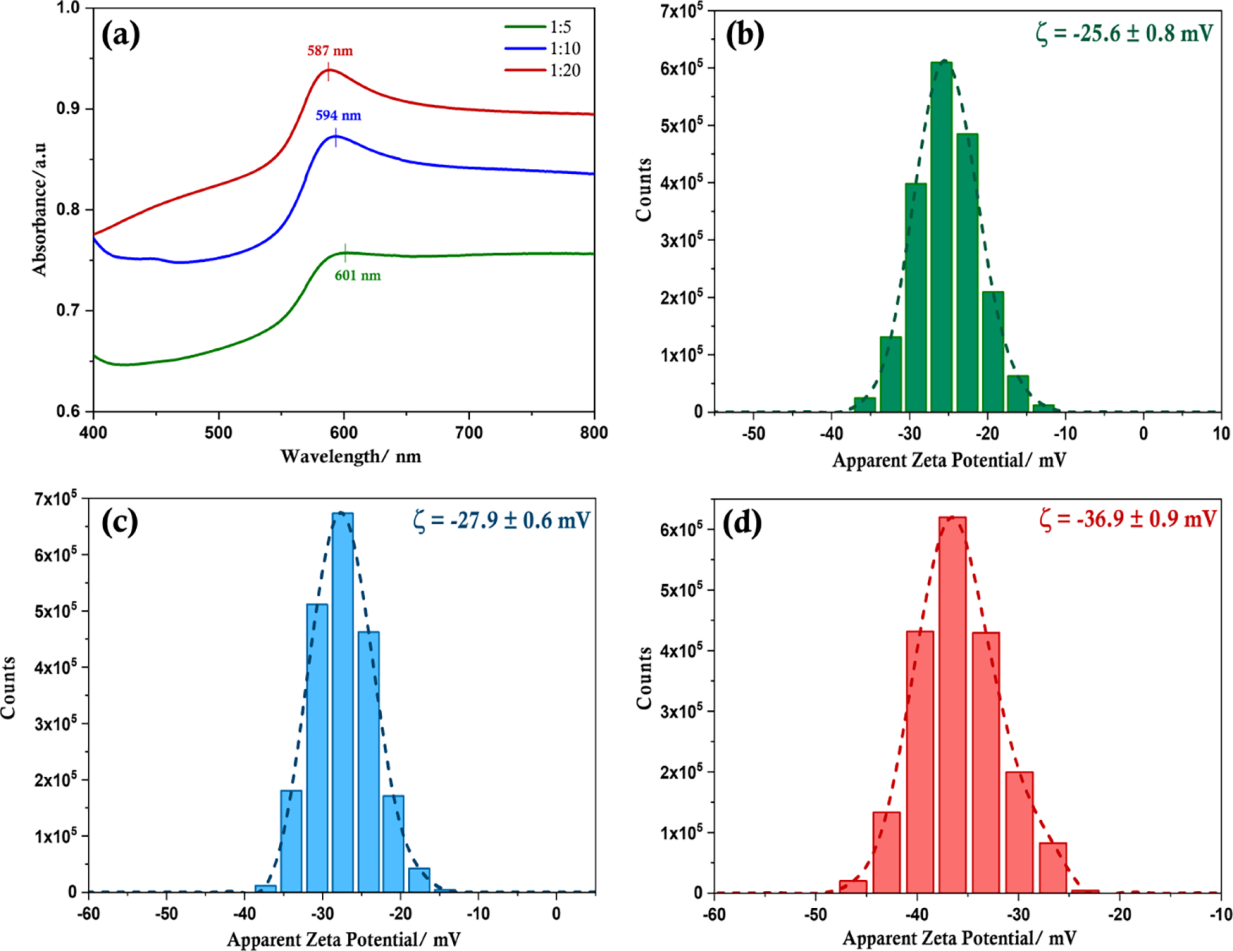
(a) UV–Vis absorption spectra of copper particles with various ratios of Cu(II): l-alanine; and zeta potential measurements of copper particles with Cu(II): l-alanine ratios of (b) 1:5, (c) 1:10, and (d) 1:20.

The trend in particle size distribution is also confirmed by UV–visible absorption spectroscopy (Figure 3a), which show that an increase in the proportion of l-alanine leads to a shift in the characteristic plasmon peak toward a shorter wavelength. Cu-Ala20, with the smallest particles, exhibits a surface plasmon resonance peak maximum (λ_SPR_) at 587 nm, which shifts to 594 and 601 nm for Cu-Ala10 and Cu-Ala5, respectively. This type of size-dependent shift in the λ_SPR_ in metal nanoparticles is associated with changes in absorption and scattering of light.^41^ The decrease in the proportion of l-alanine also results in the broadening of the peak. This observation could be attributed to the presence of rods, larger particles as well as the wider range of particle sizes, and their differential contribution to absorption and scattering. An additional synthesis with an intermediate Cu(II) to l-alanine ratio of 1:15 (Cu-Ala15) was synthesized to get a better understanding on particle size distribution (Figure S5). These particles have an average particle size of ∼309 nm and a λ_SPR_ of 597 nm (Figure S6).

The interaction of the copper atoms on the surface of the particles with l-alanine has been shown to involve both the nitrogen atom of the amine and the oxygen atom of the carboxylate group in vacuum conditions.^42^ However, the change in pH (from pH 10 to pH 4–5 upon completion of the reaction) caused by introducing the reducing agent could also affect the interaction between copper and the capping ligand. In order to elucidate the binding interactions between l-alanine and copper, zeta potential (ζ) analysis was done, which showed that the potential at the slipping plane was negative, and the magnitude of the zeta potential increased with the amount of l-alanine (Figure 3b–d and Figure S7). The negative charge is indicative of free COO^–^ groups, which are not bound to copper. A higher magnitude of the measured zeta potential is associated with a more stable suspension where there is an appreciable degree of electrostatic repulsion between adjacent particles ensuring they remain suspended in the medium. Small zeta potentials indicate stronger attractive forces, which outweigh repulsive forces causing a disruption in suspension stability, resulting in subsequent flocculation. A zeta potential of at least ±30 mV is reported to indicate a stable suspension stabilized exclusively by electrostatic repulsion.^43^ For Cu-Ala5 and Cu-Ala10, the zeta potential is slightly below −30 mV (−25.6 ± 0.8 and −27.9 ± 0.6 mV, respectively) but reaches −36.9 ± 0.9 mV for Cu-Ala20, demonstrating increased stability resulting from improved electrostatic repulsion. Additionally, the dried powder showed no aggregation or clump formation and could be re-suspended with no apparent difference in properties following 5 months of storage.

**Figure 4:**
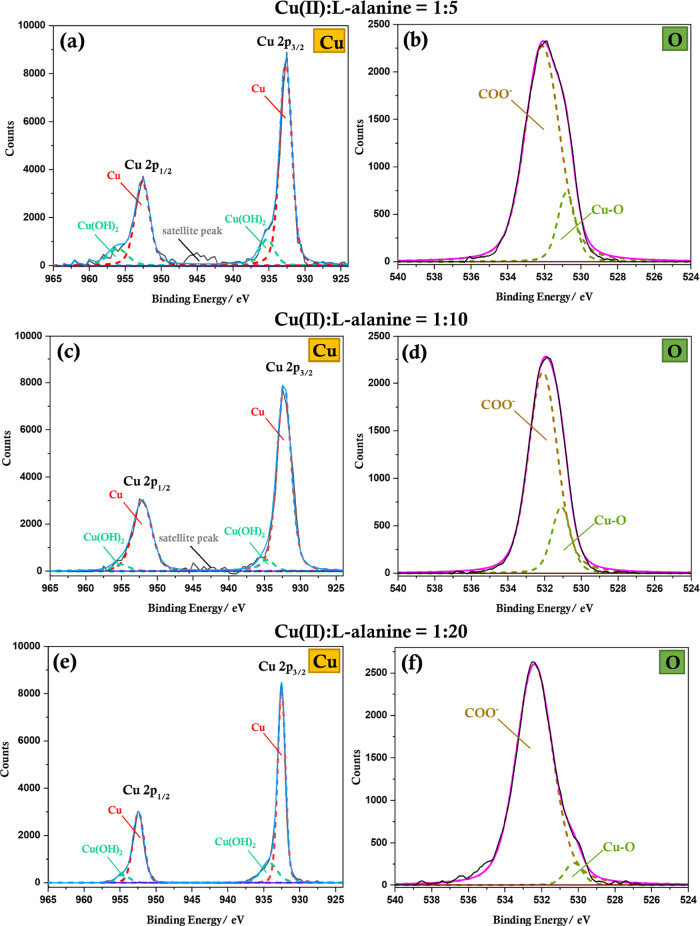
XPS measurements and peak assignments corresponding to Cu 2p (a,c,e) and O 1s (b,d,f) core levels for copper particles synthesized with Cu(II):l-alanine ratios of 1:5 (a,b); 1:10 (c,d); and 1:20 (e,f).

The XPS data (Figure 4) clearly confirm the presence of Cu(0) with 2p_3/2_ and 2p_1/2_ peaks at 932.56 ± 0.05 and 952.52 ± 0.05 eV, respectively, for all l-alanine ratios.^44^ Copper LMM Auger spectral peaks (Figure S8) observed at a kinetic energy range of 918.64–918.78 eV (corresponding binding energy range is 567.47–567.75 eV) corroborates the presence of Cu(0).^44^ For Cu-Ala5, a weak satellite peak indicative of copper oxides and hydroxides is visible together with evidence of Cu(OH)_2_ (934.65–935.25 and 954.55–956.05 eV).^44^ Importantly, as the amount of l-alanine increases, the intensity of the satellite peaks and the amount of surface hydroxides gradually decrease and oxides/hydroxides are extremely low for the Cu-Ala20 sample. This observation is also confirmed by XPS in the oxygen region, which indicates a peak in the range of 530.2–531.3 eV, which corresponds to Cu_2_O and/or Cu(OH)_2_,^4,44^ and the intensity of this peak decreases as the amount of l-alanine increases. If the more stable higher oxide, CuO, was present, a peak at a slightly lower binding energy of 529.68 ± 0.05 eV is expected.^44^ Cu(OH)_2_ could arise from the direct reaction of trace Cu(II) with NaOH, as it is a strong base. Alternatively, Cu(OH)_2_ could indicate a metastable state of oxidation observed in the presence of water/moisture, which subsequently leads to the formation of the more stable oxide, CuO.^4,45^ Cu_2_O is an oxidation product resulting from the reaction of Cu(0) with adsorbed oxygen due to electron transfer from Cu(0) to oxygen, driven by the difference in Fermi levels.^4,46^ Therefore, in the presence of both water and oxygen, Cu(OH)_2_, Cu_2_O, and CuO are all possible oxidation products;^4,46^ thus, considering that all experiments have been conducted under ambient atmospheric conditions in an aqueous medium, the amount of surface oxides present is significantly low, indicating that l-alanine plays an important role in passivating copper.

The XPS in the nitrogen (Figure S9) and oxygen regions provide more information on the nature of interaction between copper and l-alanine. The peak appearing at 400.30 ± 0.35 eV, which is also observed for pure l-alanine (Figure S10), is indicative of the nitrogen atom of the amino group.^47^ The XPS of copper particles in this region have an additional peak appearing at 397.20 ± 0.35 eV and is characteristic of a copper-nitrogen interaction, typical for nitrogen adsorbed on copper (and other transition metals).^48,49^ A third peak is observed for Cu-Ala20, which could perhaps correspond to a small proportion of free amino groups, which are not interacting with the surface atoms of copper, as these particles contain the highest amount of l-alanine. As reported for XPS of other amino acids, an asymmetry is observed at the high energy end of the peak (corresponding to COO^–^ of l-alanine) in the oxygen XPS, which can be attributed to surface charging (Figure S10).^50^

The XPS corresponding to carbon for copper particles and pure l-alanine shows similar chemical shifts, indicating a similar chemical environment (Figure S11). The intensity of the carbon XPS is shown to increase as the proportion of l-alanine increases, indicating that Cu-Ala20 has the highest density of surface ligands. Therefore, XPS and negative zeta potential values in combination corroborate and establish that the copper particles are indeed capped with the anionic form of l-alanine where the amino group is interacting directly with the surface atoms of the copper particles, while the carboxylate end, which remains uncoordinated to the surface, facilitates the formation of a stable suspension. Additionally, l-alanine is believed to be located mainly on the surface of the particles because of the low intensity of STEM-EDS response to carbon at their center and because (as will be shown later) the resulting low-temperature sintered films show very high conductivity.

**Figure 5:**
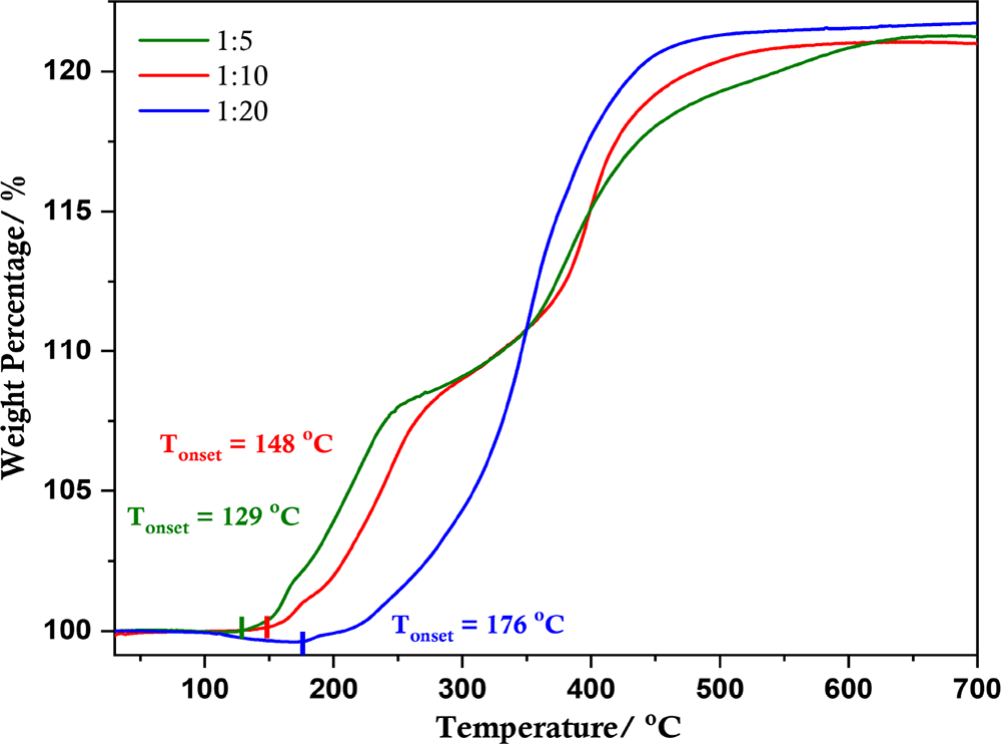
TGA curves (in air) of synthesized copper particles with various Cu(II):l-alanine ratios.

The thermal properties of l-alanine-capped copper particles were studied using TGA carried out in air (Figure 5). As the TGA was performed in air, a weight gain is observed because of the oxidation of copper with the gradual increase in temperature. Therefore, organic matter removal is not evident because of the cumulative effects of decomposition of l-alanine and gradual oxidation of copper in the temperature range of 125–250 °C. Considering the Cu-Ala5 sample, the first step in weight gain observed at ∼250 °C can be associated with the formation of Cu_2_O followed by a relatively slow increase in weight between 250 and 360 °C, which is possibly related to the formation of a metastable Cu_3_O_2_ phase observed during low-temperature oxidation of copper.^51,52^ Subsequently, beyond a temperature of ∼360 °C, the formation of CuO occurs. Interestingly, as the proportion of l-alanine increases, there is a rise in the temperature at which oxidation commences. The onset of oxidation for Cu-Ala5, Cu-Ala10, and Cu-Ala20 samples occurs at 129, 148, and 176 °C, respectively, demonstrating increased thermal stability. The onset of oxidation for Cu-Ala20 approaches the temperature at which pure l-alanine begins to decompose (Figure S12), suggesting that Cu-Ala20 has a very good compact coverage of the capping agent. It is also noted that, as the onset temperature of oxidation increases, the two distinct steps of oxidation corresponding to the formation of Cu_2_O and CuO become less sharp such that the metastable state of Cu_3_O_2_ is absent for the copper particles with the highest amount of l-alanine, suggesting the rapid formation of Cu_2_O and subsequently CuO when oxidation commences at an elevated temperature.

**Figure 6:**
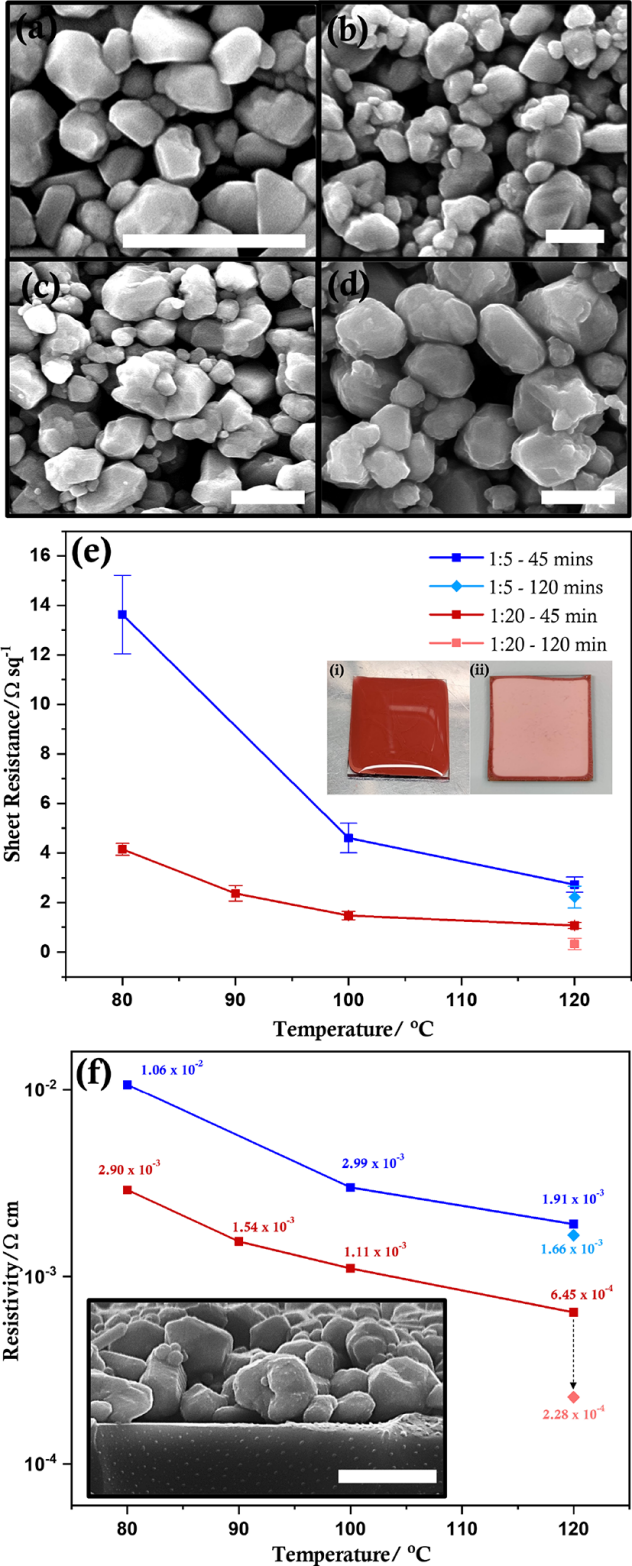
Representative SEM images of copper particles with a Cu(II):l-alanine ratio of 1:20 thermally sintered at (a) 80 °C for 45 min, (b) 100 °C for 45 min, (c) 120 °C for 45 min, and (d) 120 °C for 120 min. (e) Sheet resistance and (f) resistivity of copper films (measured at room temperature) sintered at different temperatures. Inset in panel (e): photograph of copper films before (i) and after (ii) thermal sintering; inset in panel (f): side-view SEM image of a sintered copper film showing the underlying glass substrate (bottom) and copper particles (top). The scale in all SEM images corresponds to 1 μm.

The copper particles were dispersed in a mixed solvent containing absolute ethanol and aqueous l-ascorbic acid and drop-cast on glass substrates followed by sintering in a hot vacuum desiccator. A small amount (15 mM) of l-ascorbic acid was introduced as it has been proven to provide self-reduction and self-protection properties when mixed with the copper ink paste,^53^ mitigating temperature-induced oxidation.^32^ There is a significant reduction in the measured sheet resistance (*R*_sh_) and resistivities as the sintering temperature is increased from 80 to 120 °C (Figure 6). As the temperature is gradually increased, the solvent and organics are displaced leading to particle interdiffusion and coalescence, which can be observed in the SEM images of the sintered films (Figure 6a–d). At 80 °C, small particles corresponding to the size obtained for Cu-Ala20 are observed with negligible fusion, but as the temperature increases to 120 °C, the proportion of larger particles increases, indicating continuous growth. When the sintering time was extended to 2 h, a further decrease in *R*_sh_ is observed. To provide evidence for the potential use of these particles in the fabrication of flexible conductive films, copper films (Cu-Ala20) were fabricated on a flexible polyethylene terephthalate substrate, yielding an *R*_sh_ of 1.77 ± 0.33 Ω sq^–1^ (Figure S13). Importantly, the films were compact and did not crack or delaminate from the substrate surface. This is clearly visible from the films fabricated on flexible PET substrates, which do not crack or disintegrate when distorted or bent (Figure S13).

The resistivities of the copper films are higher than that of bulk metallic copper (1.7 × 10^–6^ Ω cm), and this observation is expected as a sintering temperature of ≤120 °C is inadequate to achieve complete fusion of the copper particles. However, it is important to note that the copper films fabricated using the Cu-Ala20 particles show lower resistivity compared to other copper structures sintered at this temperature range (≤120 °C).^18,21,54−57^ Furthermore, some reports use a mix of either copper micro and nanoparticles or copper salts and particles to achieve conductivity at a lower temperature,^21,58−60^ whereas our work utilizes only the synthesized submicron copper particles and annealing has been performed at significantly lower temperatures than other studies that report low-temperature sintering.^20,59,61^ The observed conductivity at this low temperature could be attributed to the fact that the submicron copper particles synthesized in this work are capped with a compact layer of l-alanine ligands and, as proven by XPS studies, the l-alanine ligands interact with the surface atoms of copper by a Cu–N bond, which leaves the carboxylate end of the ligand free to facilitate solvation. The ink formulation uses water as part of the solvent system; therefore, the surface-anchored l-alanine ligands can also interact with water through favorable non-covalent interactions of the carboxylate group. The nature of the interaction, structural parameters, and energetics associated with the l-alanine–water interaction may vary depending on how many water molecules solvate a single ligand.^40^ This interaction also favors solvation, enabling the formation of a stable ink, which is further assisted by mixing with ethanol. Subsequently, when the ink is drop-cast on substrates and subjected to heat under vacuum during sintering, the water molecules are gradually removed by evaporation. The evaporation of water desolvates the l-alanine capping ligands, reducing the effective distance between two adjacent particles, thus, promoting coalescence and probably interaction between ligands from neighboring particles resulting in local destabilization of the capping layer, which leads to gradual growth in their size. The growth of particles may also be facilitated by particle diffusion, which reduces porosity and densifies the film. Furthermore, as the particles are of a size range, good packing is achieved with fewer pores, resulting in good contact between adjacent particles. Therefore, although this relatively low temperature (*T* ≤ 120 °C) is insufficient to completely sinter the submicron-sized copper particles, the particles are sufficiently in contact with surrounding particles (as l-alanine is a small ligand) for electron transport, rendering the films conductive at significantly lower temperatures than other systems. The existence of a mix of particle sizes contributes toward enhancing the packing density, which minimizes cracking^59^ and also facilitates low-temperature sintering.^21^

**Figure 7:**
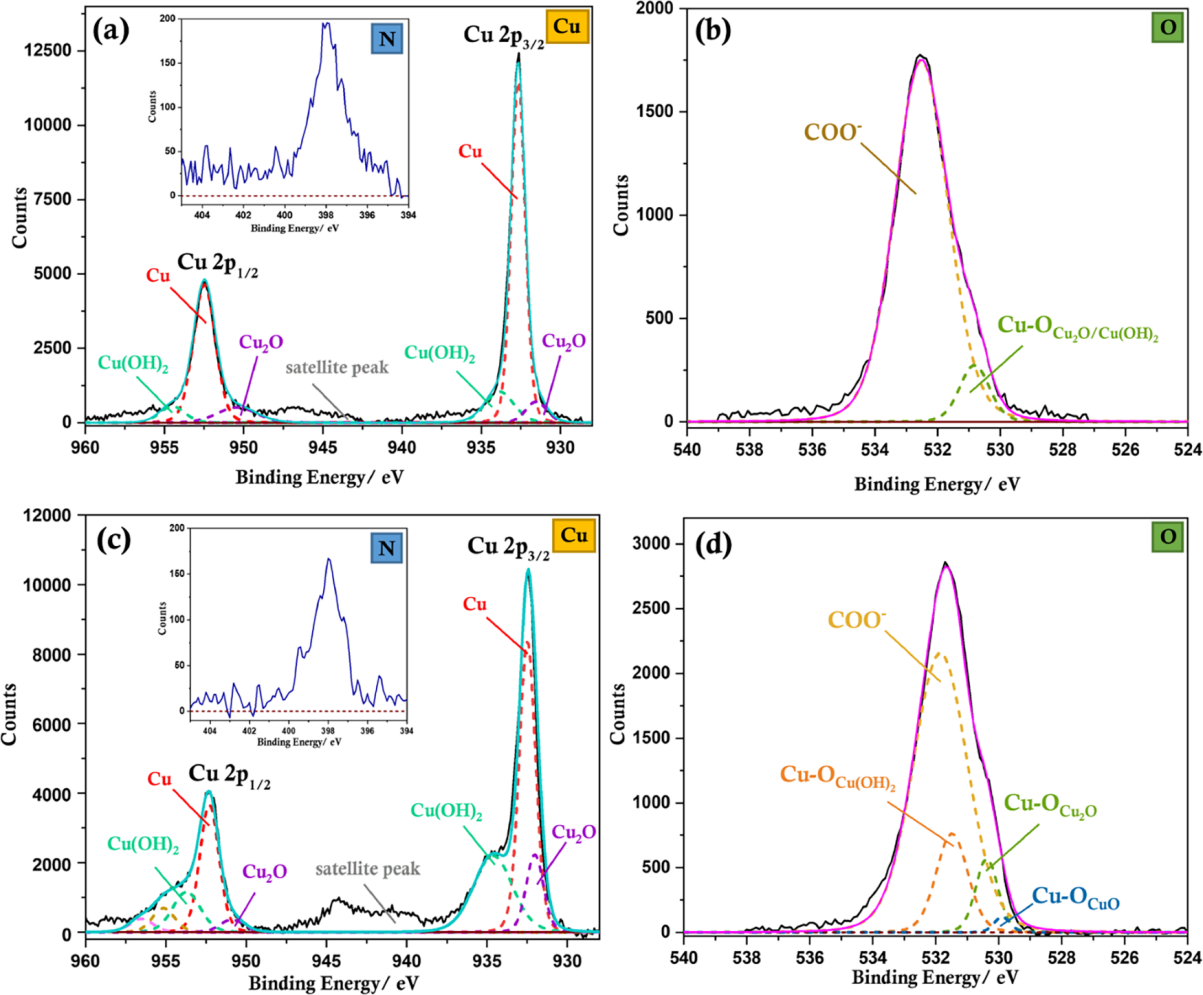
XPS measurements and peak assignments corresponding to (a) Cu 2p and (b) O 1s core levels for conductive copper films prepared with copper particles with a Cu(II):l-alanine ratio of 1:20, sintered at 120 °C for 120 min under vacuum at *t* = 5 days and (c) Cu 2p and (d) O 1s core levels for conductive copper films for *t* = 5 months after fabrication. Insets of panels (a) and (c) represent N 1s XPS measurements corresponding to *t* = 5 days and *t* = 5 months, respectively.

This observed conductivity could be accredited to the relatively low amount of surface oxides on the synthesized particles which were used to form the films as well as low surface oxides observed following sintering (Figure 7 and Figure S14). As the film is electrically conductive at all sintered temperatures, it is evident that the residual capping layer of l-alanine on the surface of the particles following sintering is sufficiently thin such that electrons can move between adjacent particles. It is also evident from the XPS corresponding to the oxygen region (Figure 7b, d and Figure S15) that the copper film formed using particles synthesized with a higher amount of l-alanine (Cu-Ala20) has undergone significantly less oxidation. Therefore, the combined influence of both l-ascorbic acid, which is known for its antioxidant properties,^32^ and l-alanine successfully mitigates the oxidation of copper, facilitating the fabrication of relatively highly conductive copper films *via* thermal sintering at a significantly lower temperature than present systems.

To study the effect of l-alanine on the long-term stability of the copper films, they were stored under ambient conditions for 5 months and XPS measurements were conducted to evaluate the degree of surface oxidation caused by atmospheric oxygen and moisture. As shown in Figure 7c, although oxidation is inevitable under these conditions, resulting in an increase in the amount of surface hydroxides and oxides (Cu_2_O and CuO), the degree of oxidation is remarkably small considering the time period exposed to air. The proportion of surface hydroxides (and Cu_2_O) is notable compared to the higher oxide, CuO, indicating early stages of oxidation.^4^ Therefore, it is evident that the layer of l-alanine capping molecules present on the copper surface is adequately compact to provide highly effective passivation enabling significant reduction in surface oxidation during long-term exposure to atmospheric oxygen and moisture yet is sufficiently thin enough such that the particles are not electrically isolated from one another, facilitating the formation of electrically conductive films at low temperature.

To further establish that the surface l-alanine molecules do not adversely interfere and affect the inherent properties of copper, the catalytic activity of annealed copper films was tested. For this purpose, the catalytic reduction of 4-nitrophenol to 4-aminophenol was used as it is a well-known and trusted model to study the catalytic activity of metal particles.^62^ A flexible sintered copper film of dimensions 4 × 4 mm was used to reduce 4-nitrophenol to 4-aminophenol. Upon completion of the reaction, the copper film was carefully removed, washed with deionized water, and used in a subsequent reaction. This process was repeated for five successive cycles.

**Figure 8:**
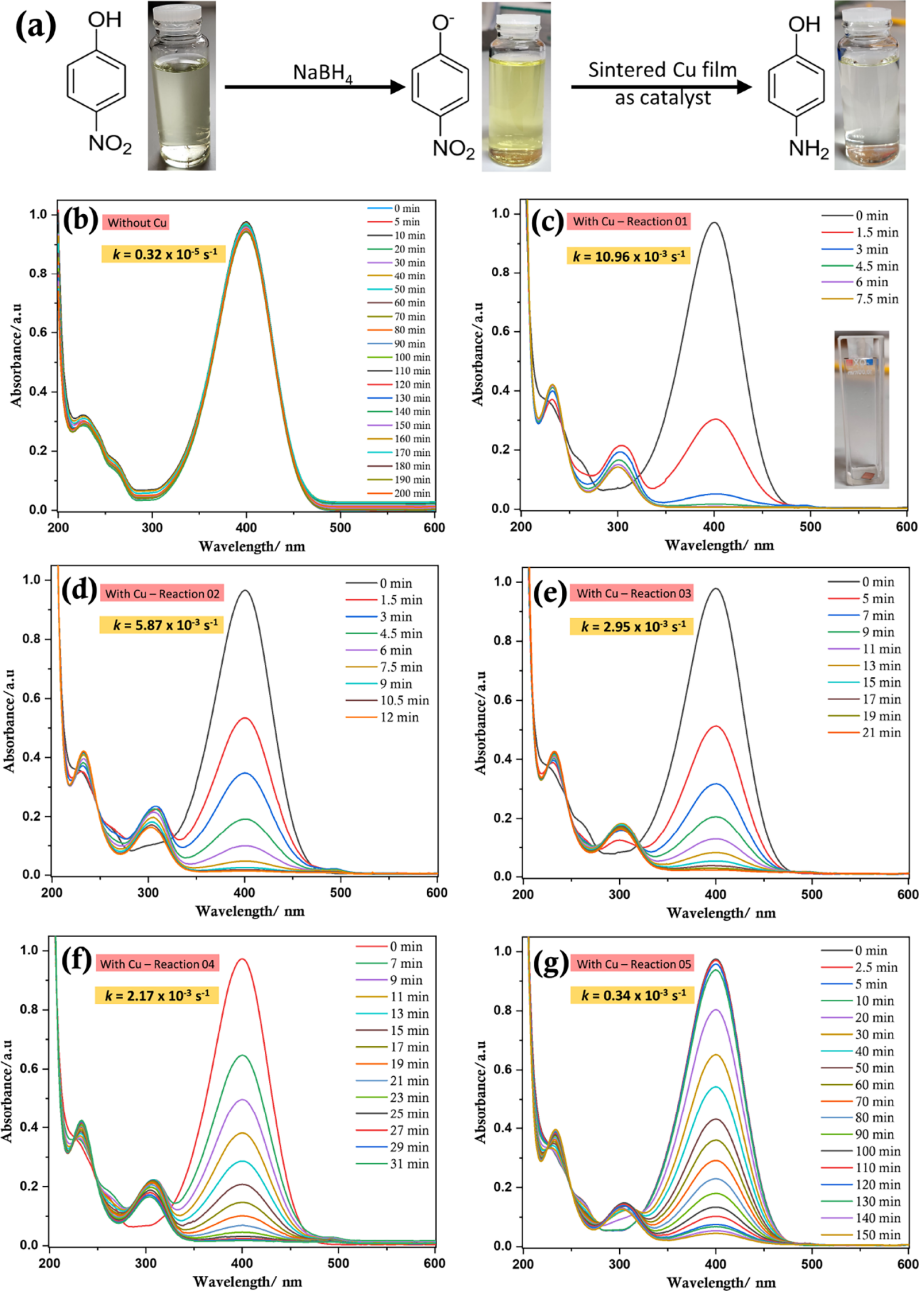
(a) Reaction scheme showing the catalytic conversion of 4-nitrophenol to 4-aminophenol in an aqueous medium under ambient conditions; UV–Vis spectra showing the progress of the reaction over time in the (b) absence of copper and (c–g) presence of copper, in five consecutive reaction cycles. Inset of panel (c) shows a photograph of the 4 × 4 mm annealed flexible copper film (placed in the cuvette) used for the catalytic conversion.

As shown in Figure 8, at all reaction cycles, the rate of the reaction is significantly faster (∼3.5 × 10^3^ times for Reaction 1 and ∼1.0 × 10^2^ times for Reaction 5) than in the absence of a catalyst. Interestingly, the rate of the reaction for the first cycle was higher than similar reports in the literature that utilize various copper nanoparticles^63,64^ and comparable with the rates obtained by Yang *et al.*(37) Although the reaction rate decreases with successive reaction cycles, even the slowest reaction is two orders of magnitude faster than the reaction in the absence of copper. Hence, it is evident that the l-alanine-capped copper films are not only extremely effective in reducing 4-nitrophenol to 4-aminophenol but also very efficient as a reusable catalyst. This result corroborates the postulate that residual l-alanine present on the surface of the copper film does not impede certain surface properties inherent to copper. Notably, it may be possible to further increase the reusable catalytic activity by washing the film with l-ascorbic acid or glacial acetic acid (instead of deionized water) to remove any surface oxides, which would enable the achievement of an improved rate in successive cycles, (i.e., minimize the reduction of the reaction rate in consecutive reactions). The latter, glacial acetic acid, is specifically known to selectively remove copper oxides.^65^

### Green Chemistry Comparison with Existing Protocols

Hydrazine hydrate, which is the predominantly used reducing agent in the synthesis of copper particles, is industrially produced by four main methods: (i) the Raschig process, which is the original method by which hydrazine was produced, based on the oxidation of ammonia using sodium hypochlorite; (ii) the Schestakov synthesis where urea is used as the nitrogen source; (iii) the Bayer process in which ammonia is oxidized in the presence of an aliphatic ketone, resulting in the formation of ketazine, which is subsequently hydrolyzed; and (iv) the Pechiney–Ugine–Kuhlmann or peroxide-ketazine process where hydrogen peroxide is used to oxidize ammonia in the presence of a ketone.^66^ All these processes are highly energy-intensive. There are many concerns in all these processes, especially the production of a large amount of chlorine byproducts and the instability of chloramine and hydrazine in (i) and (ii).^67^ Moreover, as a result of the combustible nature of hydrazine, extreme care must be taken to prevent accidents. Conversely, the benign l-ascorbic acid used in this work is industrially produced by modification of the classical Reichstein process using d-glucose as the precursor with over 90% yield in each step.^68^ Furthermore, most of the l-ascorbic acid produced globally is made by the fermentative oxidation of l-sorbose using *Ketogulonicigenium vulgare*.^68^ The stabilizing and capping agent used in this work, l-alanine, is traditionally produced by the enzymatic conversion of l-aspartic acid, which utilizes immobilized microbial cells.^69^ However, similar to the industrial production of many amino acids, the production of l-alanine by fermentation is preferred owing to the renewable and inexpensive nature of feedstock sugars such as d-glucose. Additionally, many improvements in the latter have been reported by careful engineering of microorganisms used in the fermentation process.^69^

Therefore, our approach to synthesize and fabricate copper films uses nontoxic chemicals and solvents, reduces waste generation, and promotes safer chemistry than existing synthesis methods while also producing highly stable copper particles and conductive films by means of low-temperature sintering.

## Conclusions

A green, low temperature, aqueous synthetic approach for the preparation of submicron-sized copper particles under ambient atmospheric conditions using l-alanine as the capping agent provides a less hazardous and easy route to the colloids compared with existing approaches. The ratio of copper precursor to amino acid influences significantly the size, shape, and susceptibility of particles towards oxidation. It is demonstrated that high proportions of capping ligand give smaller particles with excellent stability toward ambient and thermal oxidation. The copper particles attain stability partially through a copper–nitrogen interaction between the metal and the amino acid. The small size of the capping ligand provides adequate barrier properties to the particles without disrupting electron transport dramatically, ensuring the formation of a conductive film when sintered at a low temperature (≤120 °C) compared with other systems. The residual l-alanine capping molecules on the surface of the copper facilitate long-term passivation of sintered metal films, mitigating oxidation under normal atmospheric conditions without negatively impacting the surface properties. To prove this property, the copper films have been employed as effective and reusable catalysts in the reduction of 4-nitrophenol to 4-aminophenol.

The use of chemicals and solvents that are more benign and sustainable than those used habitually for copper inks and the low-temperature processing conditions together with the synergistic combination of favorable technical features make these copper particles potentially beneficial to existing materials in a number of applications. Particularly, as demonstrated, the high conductivities achieved at relatively low temperatures would be beneficial to the flexible electronics industry because the tolerance of flexible substrates to high processing temperatures is inadequate.

## Supporting Information Available

Experimental details, characterization details, structure characterization (optical microscopy images, TEM images, STEM-EDS elemental maps, SAED and XRD patterns), UV–Vis absorption spectra, zeta potential measurements, and XPS of sintered copper films (PDF)
