# Supplementary material for: Low-Temperature Sintering of l-Alanine-Functionalized Metallic Copper Particles Affording Conductive Films with Excellent Oxidative Stability
Source: ACS Appl Electron Mater. 2022 May 3;4(5):2502–15. doi: 10.1021/acsaelm.2c00275 (PMC9134346; doi:10.1021/acsaelm.2c00275)
Supplement: Supplementary file 1 — el2c00275_si_001.pdf [file el2c00275_si_001.pdf]

Supporting Information

For

Low-temperature sintering of L-alanine functionalized metallic copper particles affording conductive films with excellent oxidative stability

H. Jessica Pereira[1*], C. Elizabeth Killalea[1†] and David B. Amabilino[1‡]

[1]The GSK Carbon Neutral Laboratories for Sustainable Chemistry, School of Chemistry, University of Nottingham, Nottingham, NG7 2TU, United Kingdom.
[†]Current Address: University Libre de Bruxelles, Avenue Franklin Roosevelt 50, B -1050, Bruxelles, Belgium
[‡]Current Address: Institut de Ciència de Materials de Barcelona, Consejo Superior de Investigaciones Científicas, Campus UAB, 08193 Bellaterra, Catalunya, Spain
*Corresponding Author - Email: jessica.pereira@nottingham.ac.uk

Common choices	Hazards	Greener options
**Capping Agent**		
CTAB (Hexadecyltrimethylammonium bromide)[1,2]	H302 - Harmful if swallowed<br>H315 - Causes skin irritation<br>H318 - Causes serious eye damage<br>H335 - May cause respiratory irritation<br>H373 - May cause damage to organs through prolonged or repeated exposure<br>H400 - Very toxic to aquatic life	Chitosan[3,4]<br>Cyclodextrins[5]<br>Polyvinylpyrrolidone[6]<br>L-Cyseine[7]<br>Oleic acid[8,9]<br>L-alanine[10] (and this work)
Sodium dodecyl sulfate[6,11]	H228 - Flammable solid<br>H302 - Harmful if swallowed<br>H315 - Causes skin irritation<br>H318 - Causes serious eye damage<br>H335 - May cause respiratory irritation<br>H412 - Harmful to aquatic life with long lasting effects	
Alkyl amines (hexyl, octyl, hexadecyl)[12–14]<br>(May also serve as reducing agents)	H226 - Flammable liquid and vapor<br>H301 + H311 - Toxic if swallowed or in contact with skin<br>H314 - Causes severe skin burns and eye damage<br>H332 - Harmful if inhaled<br>H335 - May cause respiratory irritation<br>H410 + H411 - Very toxic to aquatic life with long lasting effects<br>H373 - May cause damage to organs (Gastrointestinal tract, Liver, Immune system) through prolonged or repeated exposure	
Lactic acid[9,10]	H315 - Causes skin irritation<br>H318 - Causes serious eye damage	
Glycolic acid[9,10]	H314 - Causes severe skin burns and eye damage<br>H332 - Harmful if inhaled	
Acetic acid[10]	H226 - Flammable liquid and vapor<br>H314 - Causes severe skin burns and eye damage	
1-amino-2-propanol[15]	H314 - Causes severe skin burns and eye damage	
Nitrilotriacetic acid disodium salt[16]	H302 - Harmful if swallowed.<br>H351 - Suspected of causing cancer	
**Reducing Agent**		
Hydrazine hydrate[1,9–11,15–18]	H301 + H311 - Toxic if swallowed or in contact with skin<br>H314 - Causes severe skin burns and eye damage<br>H317 - May cause an allergic skin reaction<br>H330 - Fatal if inhaled<br>H350 - May cause cancer<br>H400 - Very toxic to aquatic life	Glucose[1,7,13,14]<br>1,2-hexadecanediol[8]<br>Sodium hypophosphite[4]<br>Ascorbic acid[2,3,5,19,20]
Oleylamine[13,21]	H302 - Harmful if swallowed<br>H304 - May be fatal if swallowed and enters airways<br>H314 - Causes severe skin burns and eye damage<br>H335 - May cause respiratory irritation<br>H373 - May cause damage to organs (Gastrointestinal tract, Liver, Immune system) through prolonged or repeated exposure<br>H410 - Very toxic to aquatic life with long lasting effects	
Sodium Borohydride[17,19]	H260 - In contact with water releases flammable gases which may ignite spontaneously<br>H301 + H311 - Toxic if swallowed or in contact with skin<br>H314 - Causes severe skin burns and eye damage	
Vanadium Sulfate[6]	H301+H311+H331 - Toxic if swallowed, in contact with skin or if inhaled<br>H373 - May cause damage to organs (Gastrointestinal tract, Liver, Immune system) through prolonged or repeated exposure	
Formic Acid[22]	H226 - Flammable liquid and vapor<br>H302 - Harmful if swallowed<br>H314 - Causes severe skin burns and eye damage<br>H331 - Toxic if inhaled<br>EUH071 - Corrosive to the respiratory tract	
**Solvent/ Solvent Additives**		
Oleylamine[8,21,23]	H302 - Harmful if swallowed<br>H304 - May be fatal if swallowed and enters airways<br>H314 - Causes severe skin burns and eye damage<br>H335 - May cause respiratory irritation<br>H373 - May cause damage to organs (Gastrointestinal tract, Liver, Immune system) through prolonged or repeated exposure<br>H410 - Very toxic to aquatic life with long lasting effects	Water[2–5,7,14]<br>Alcohols[18,23]
Ethylene glycol[6,15]	H302 - Harmful if swallowed<br>H373 - May cause damage to organs through prolonged or repeated exposure	

Table S1: List of capping agents, reducing agents and solvents/solvent additives commonly used in the synthesis of copper nanostructures of different dimensions and shapes, hazards associated with these chemicals and more sustainable alternatives proven to be effective.

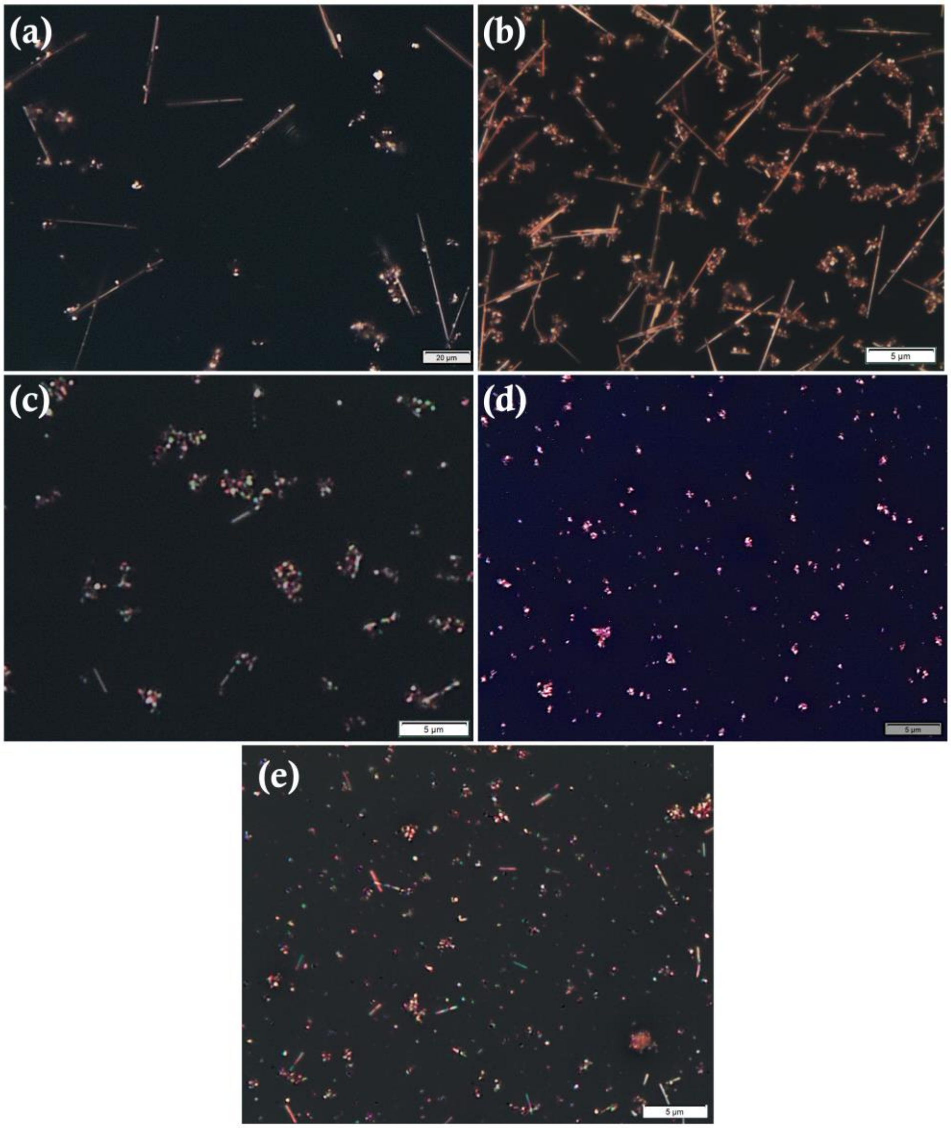

Figure S1: Optical microscope images obtained under reflection mode for copper synthesis carried out at a (a) low temperature (45 °C); (b) pH 8-9; (c) higher rate of addition of reducing agent (3 ml min^{-1}); and (d) pH > 10, at 75 °C where the reducing agent was added at a rate of 0.25 ml min^{-1} and (e) higher concentration of reducing agent (1.7 M, pH > 10, at 75 °C where addition was done at a rate of 0.25 ml min^{-1}) for a Cu(II): L – alanine ratio of 1:10.

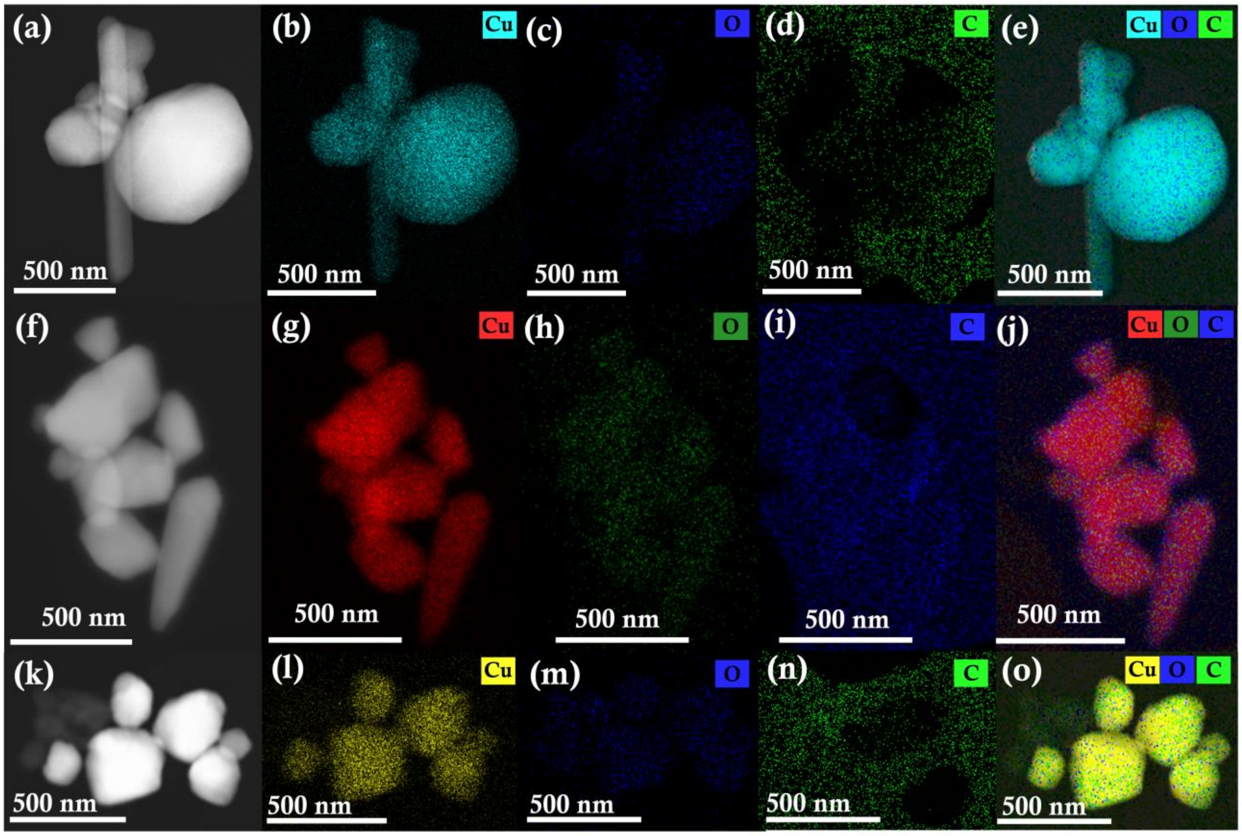

Figure S2: (i) HAADF/ADF – STEM images (a, f and k); STEM -EDS elemental maps corresponding to copper (b, g, and l), oxygen (c, h and m), carbon (d, i and n) and overlays (e, j and o) showing the distribution of all elements for copper particles with a Cu(II): L – alanine ratio of 1:5 (a - e); 1:10 (f - j) and 1:20 (k - o) respectively.

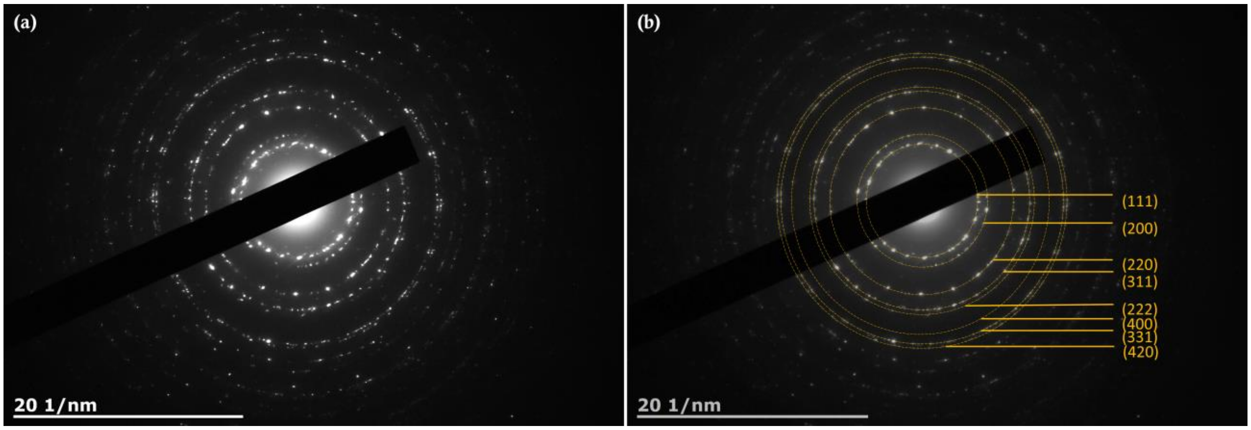

Figure S3: (a) Selected-area electron diffraction (SAED) patterns for copper particles with a Cu(II): L – alanine ratio of 1:20 and (b) annotated SAED pattern showing the crystal planes represented by each ring pattern.

Interpretation of SAED pattern and identification of d-spacings was performed using ImageJ and Mercury software. Crystal planes (hkl) have been assigned by comparing with the standard ICSD – 43493 and are in good agreement, indicating the presence of a FCC (space group: $Fm\overline{3}m$) crystal structure.

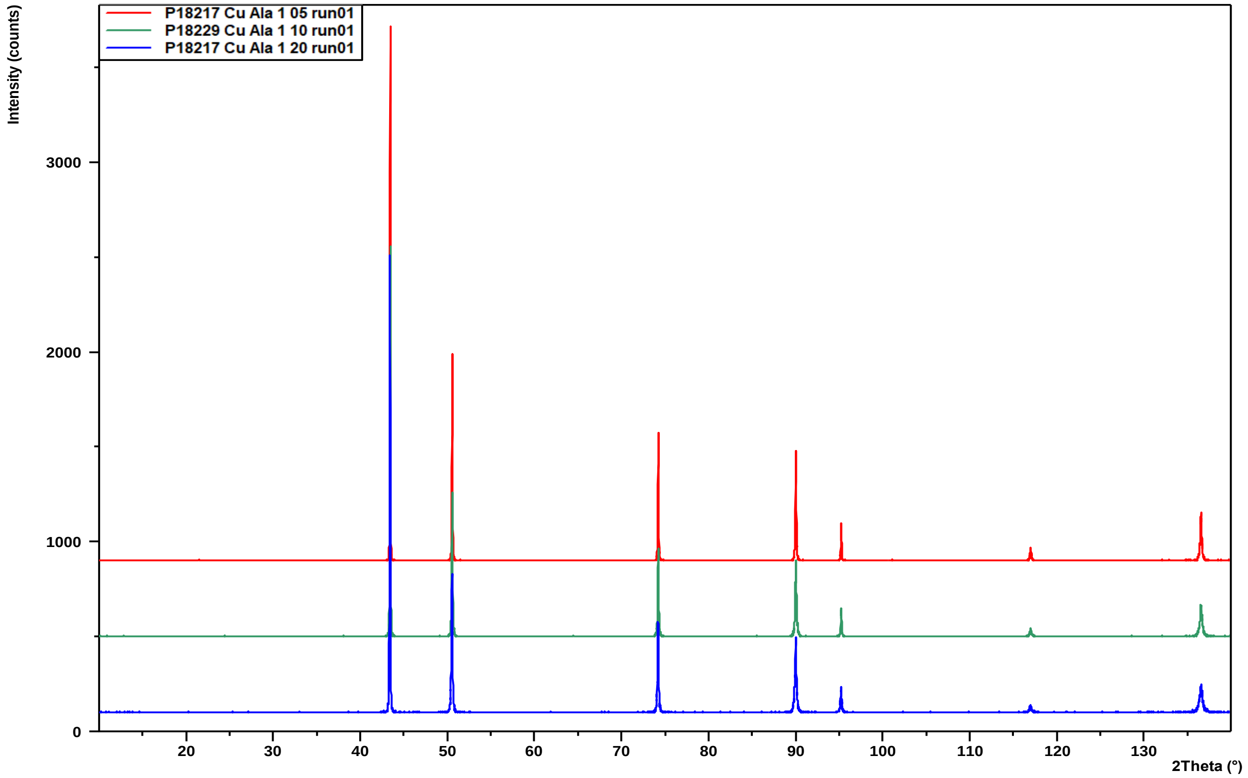

Figure S4: XRD patterns of L-alanine capped copper particles performed in air. Patterns shown in red, green and blue correspond to Cu-Ala5, Cu-Ala10 and Cu-Ala20 respectively. XRD patterns have been offset along the Y-axis for clarity.

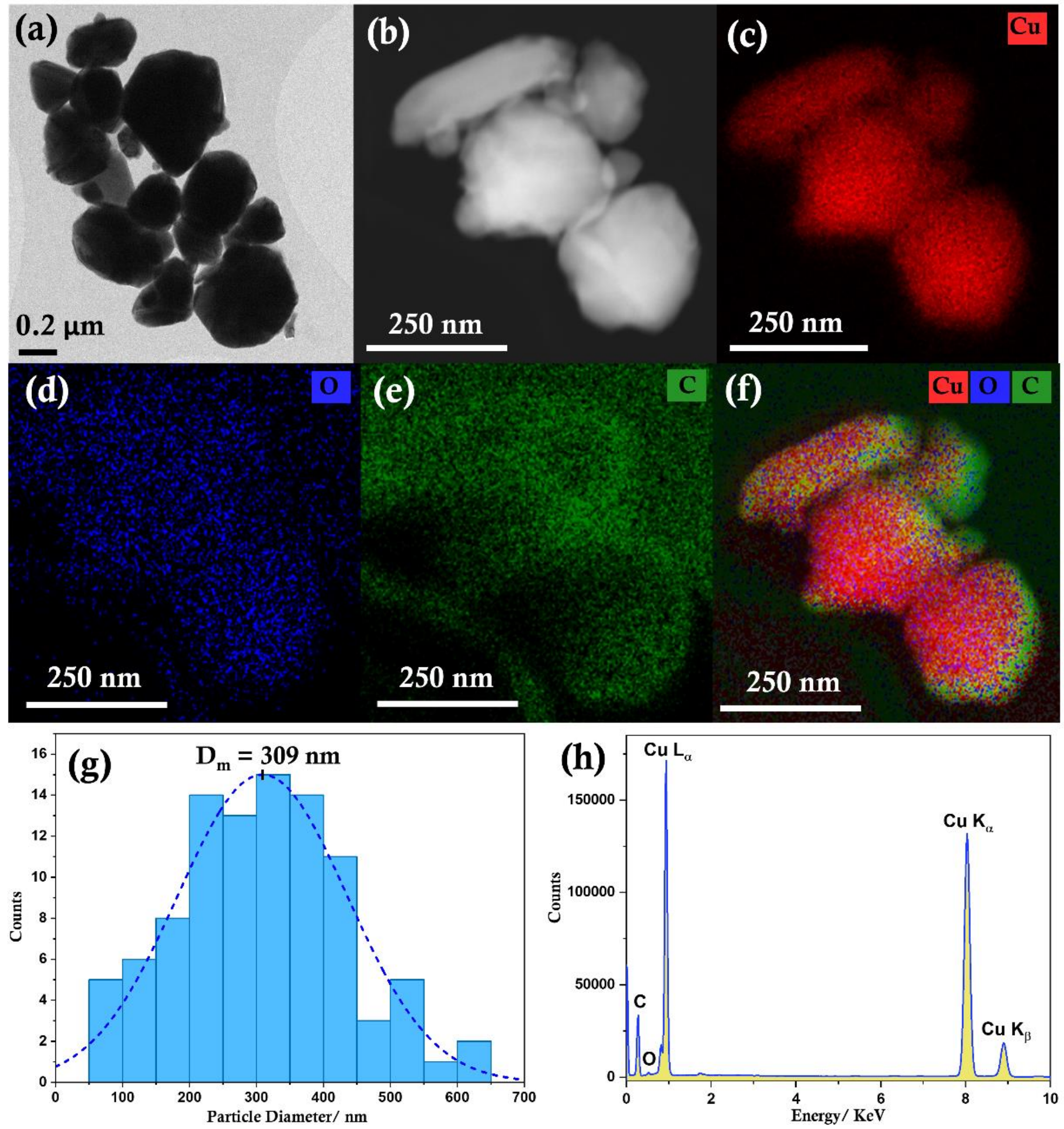

Figure S5: (a) A representative TEM image; (b) HAADF – STEM image; STEM -EDS elemental maps corresponding to (c) copper; (d) oxygen; (e) carbon; and (f) overlays showing the distribution of all elements; (g) particle size distribution and (h) EDS analysis of copper particles with a Cu(II): L – alanine ratio of 1:15 (Cu-Ala15). Particle size distribution has been computed considering at least 100 particles.

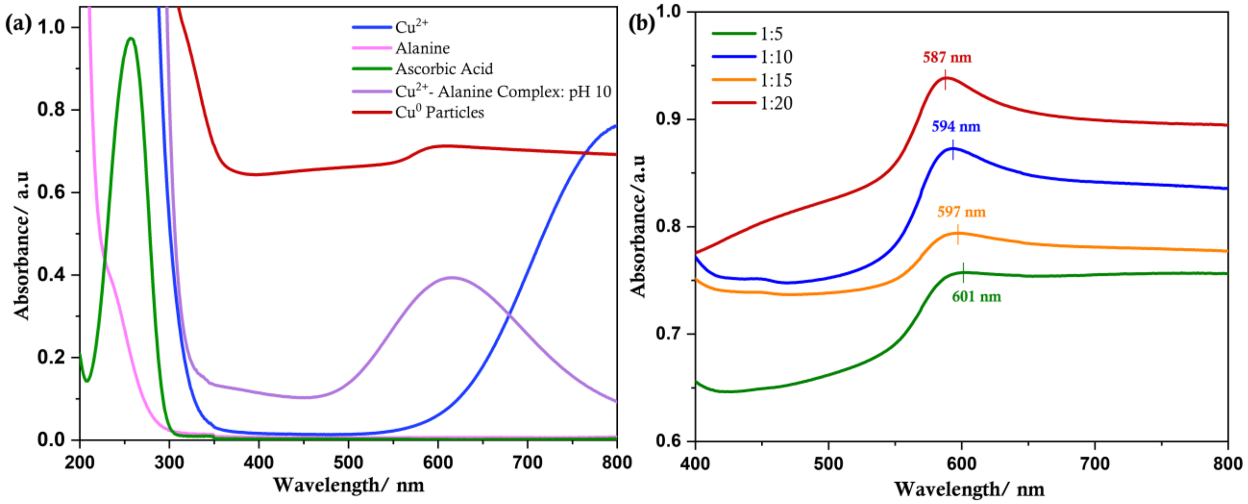

Figure S6: UV-Vis absorption spectra of (a) the reactants and products in aqueous medium; and (b) copper particles with various ratios of Cu(II):L-alanine.

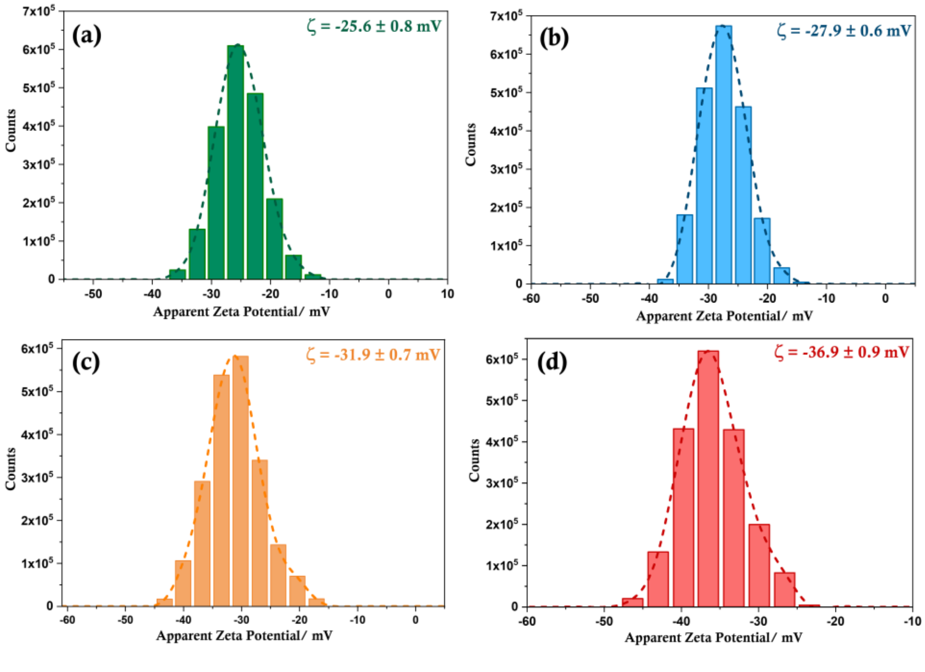

Figure S7: Zeta potential measurements of copper particles with a Cu(II):L-alanine ratio of (a) 1:5; (b) 1:10; (c) 1:15 and; (d) 1:20.

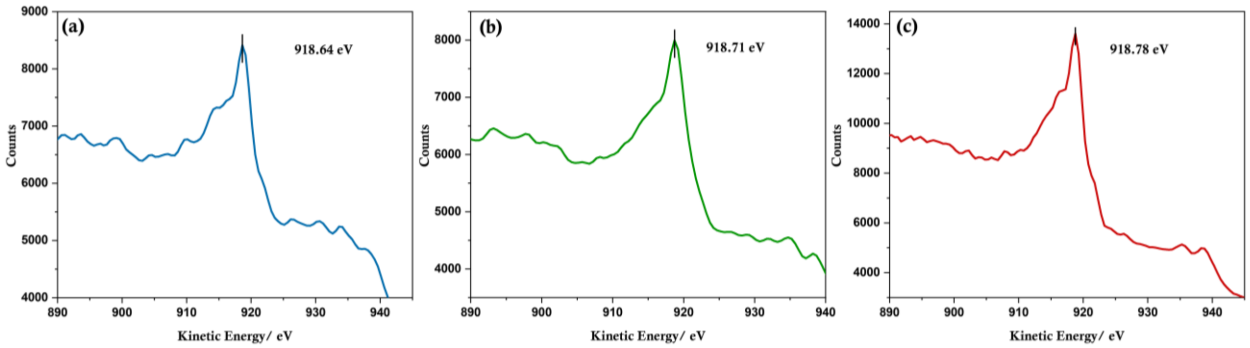

Figure S8: Cu LMM Auger spectra corresponding to copper particles with a Cu(II):L-alanine ratio of (a) 1:5; (b) 1:10; and (c) 1:20.

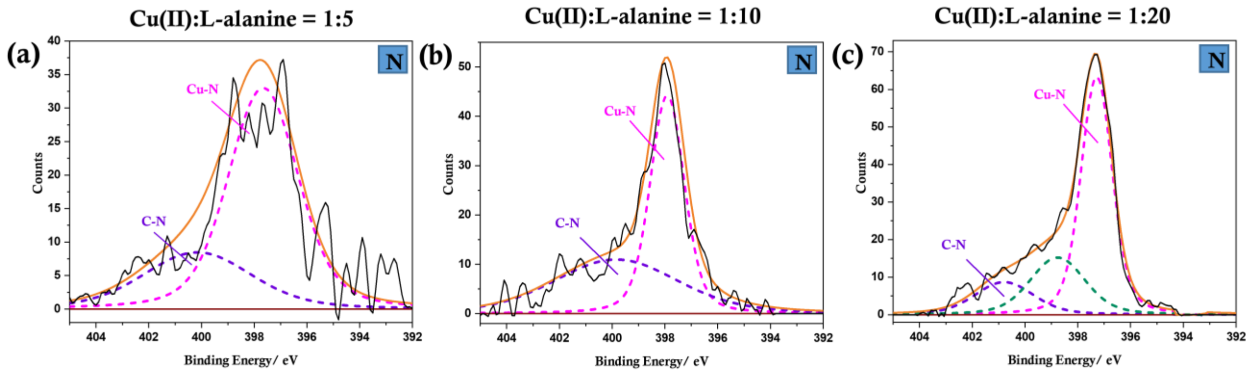

Figure S9: XPS measurements and peak assignments corresponding to N 1s core levels for copper particles synthesized with a Cu(II):L-alanine ratio of (a)1:5; (b)1:10 and (c) 1:20, respectively.

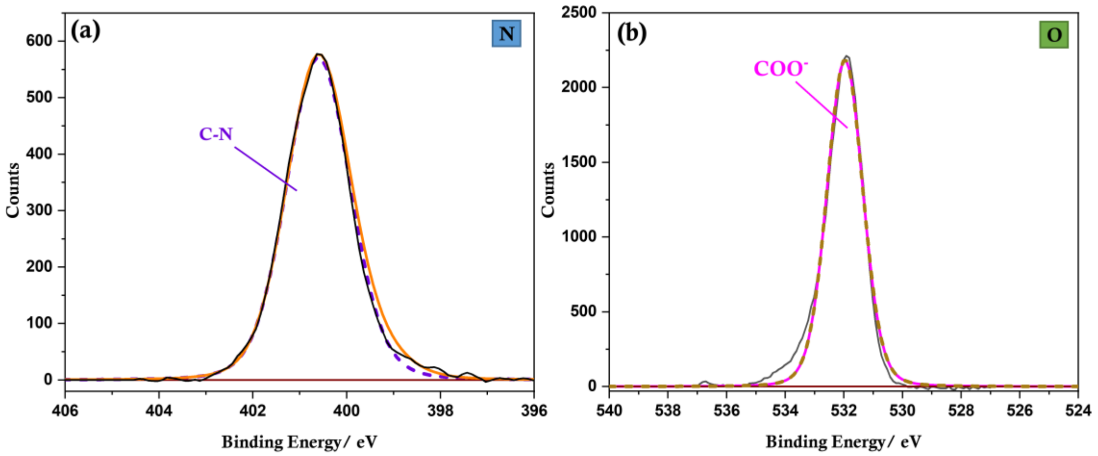

Figure S10: XPS measurements and peak assignments corresponding to (a) N 1s; and (b) O 1s core levels for L-alanine.

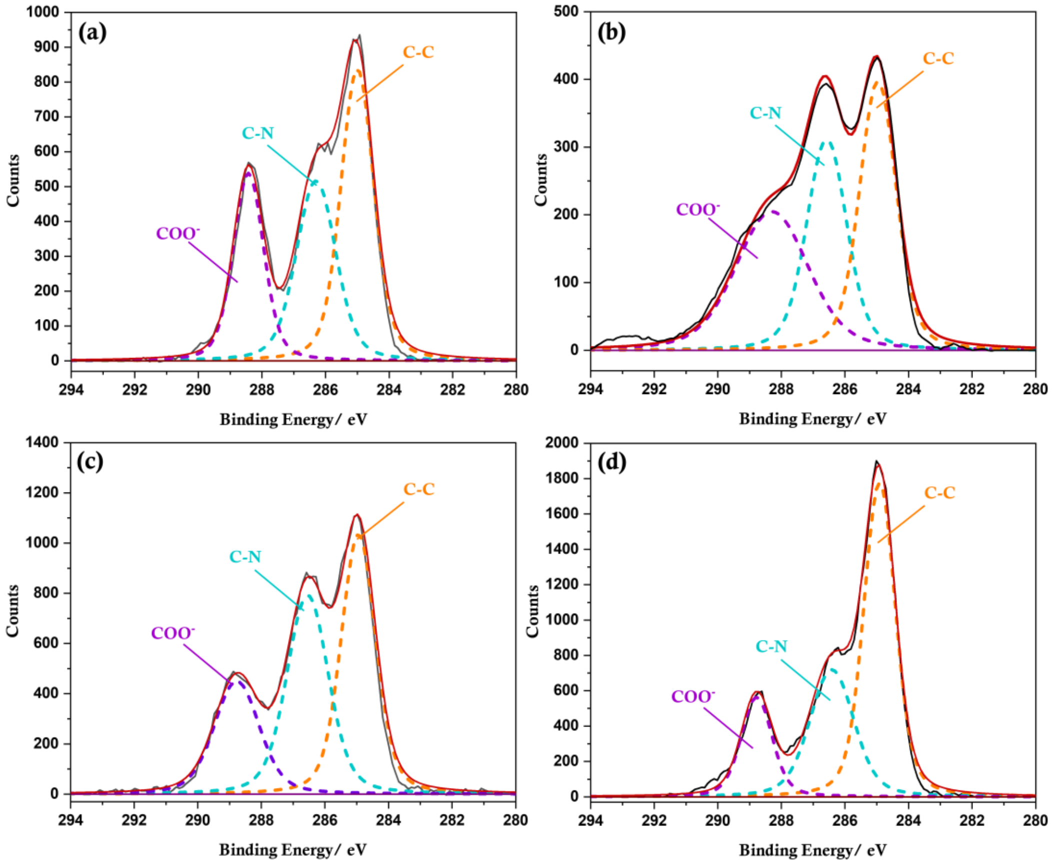

Figure S11: XPS measurements and peak assignments corresponding to C 1s core levels of (a) L-alanine; copper particles synthesized with a Cu(II):L-alanine ratio of (b) 1:5; (c)1:10 and (d) 1:20 respectively.

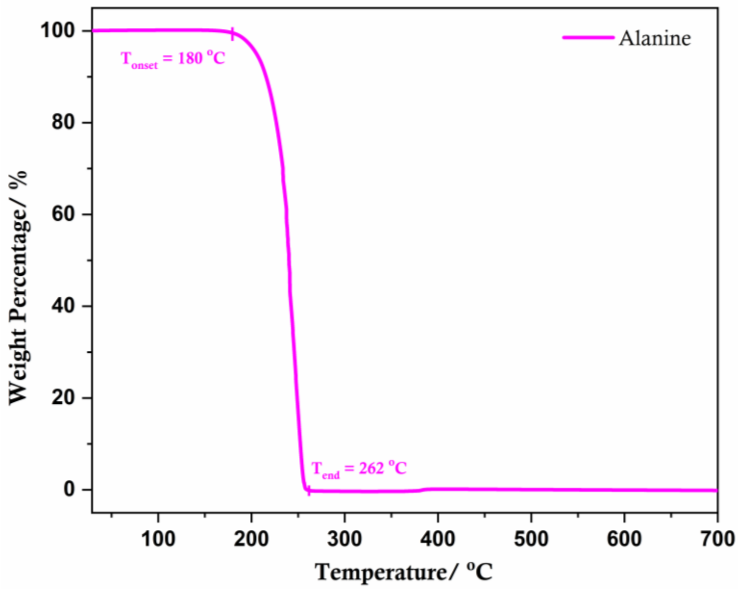

Figure S12: TGA measurement corresponding to L-alanine, performed in an inert atmosphere (N_2).

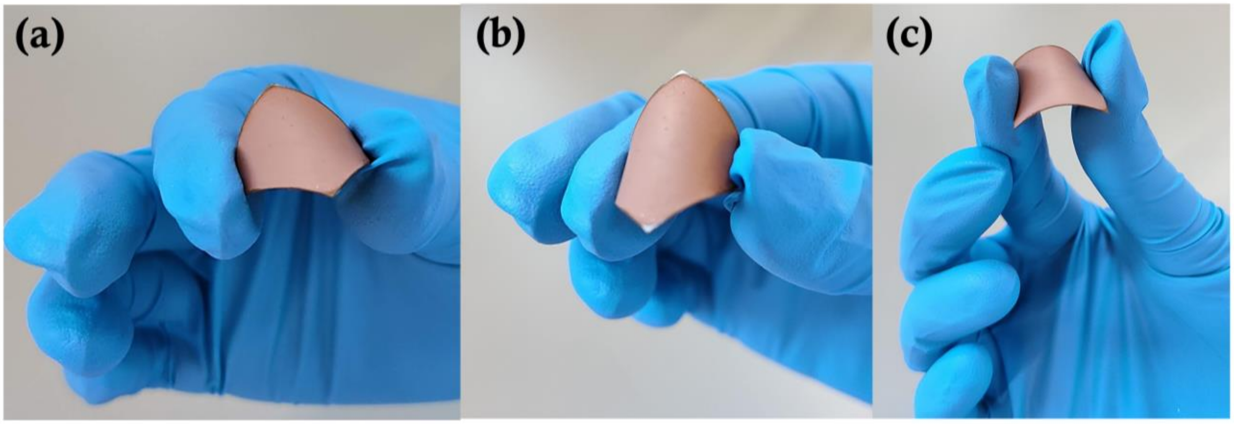

Figure S13: (a), (b) and (c) are photographs of copper films formed on a flexible poly(ethylene terephthalate) substrate using the Cu-Ala20 particles, sintered at 120 °C for 45 minutes under vacuum.

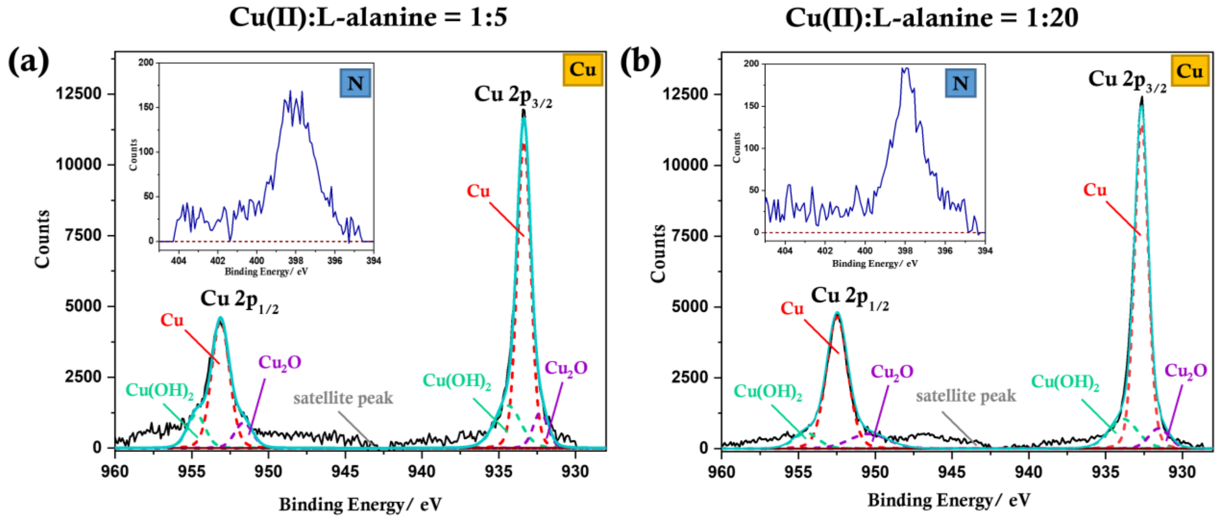

Figure S14: XPS measurements and peak assignments corresponding to Cu 2p core levels for conductive copper films prepared with copper particles with a Cu(II): L-alanine ratio of (a)1:5 and (b) 1:20, sintered at 120 °C for 120 minutes under vacuum. ***Inset:*** N 1s XPS measurements for conductive copper films prepared with Cu particles with a Cu(II): L-alanine ratio of (a)1:5; and (b)1:20, respectively.

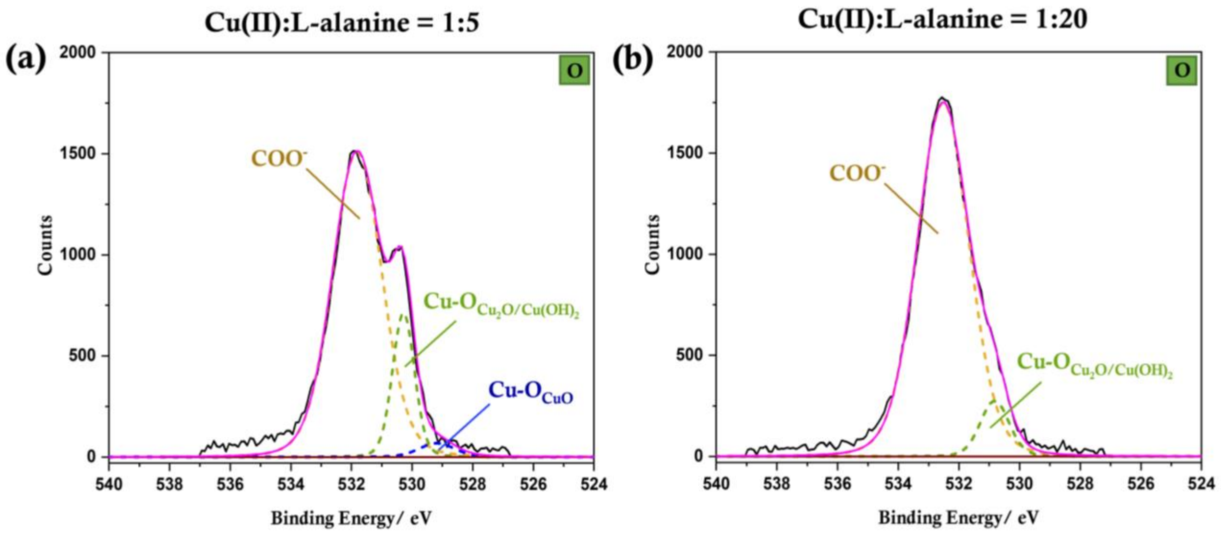

Figure S15: XPS measurements and peak assignments corresponding to O 1s core levels for conductive copper films prepared with copper particles with a Cu(II): L-alanine ratio of (a)1:5 and (b) 1:20, sintered at 120 °C for 120 minutes under vacuum.

References

(1) Granata, G.; Yamaoka, T.; Pagnanelli, F.; Fuwa, A. Study of the Synthesis of Copper Nanoparticles: The Role of Capping and Kinetic towards Control of Particle Size and Stability. *J. Nanoparticle Res.* **2016**, *18* (5), 133. https://doi.org/10.1007/s11051-016-3438-6.

(2) Biçer, M.; Şişman, I. Controlled Synthesis of Copper Nano/Microstructures Using Ascorbic Acid in Aqueous CTAB Solution. *Powder Technol.* **2010**, *198* (2), 279–284. https://doi.org/10.1016/j.powtec.2009.11.022.

(3) Zain, N. M.; Stapley, A. G. F.; Shama, G. Green Synthesis of Silver and Copper Nanoparticles Using Ascorbic Acid and Chitosan for Antimicrobial Applications. *Carbohydr. Polym.* **2014**, *112*, 195–202. https://doi.org/10.1016/j.carbpol.2014.05.081.

(4) Tokarek, K.; Hueso, J. L.; Kuśtrowski, P.; Stochel, G.; Kyzioł, A. Green Synthesis of Chitosan-Stabilized Copper Nanoparticles. *Eur. J. Inorg. Chem.* **2013**, No. 28, 4940–4947. https://doi.org/10.1002/ejic.201300594.

(5) Suárez-Cerda, J.; Espinoza-Gómez, H.; Alonso-Núñez, G.; Rivero, I. A.; Gochi-Ponce, Y.; Flores-López, L. Z. A Green Synthesis of Copper Nanoparticles Using Native Cyclodextrins as Stabilizing Agents. *J. Saudi Chem. Soc.* **2017**, *21* (3), 341–348. https://doi.org/10.1016/j.jscs.2016.10.005.

(6) Reverberi, A.; Salerno, M.; Lauciello, S.; Fabiano, B. Synthesis of Copper Nanoparticles in Ethylene Glycol by Chemical Reduction with Vanadium (+2) Salts. *Materials (Basel).* **2016**, *9* (10), 809. https://doi.org/10.3390/ma9100809.

(7) Kumar, N.; Upadhyay, L. S. B. Facile and Green Synthesis of Highly Stable L-Cysteine Functionalized Copper Nanoparticles. *Appl. Surf. Sci.* **2016**, *385*, 225–233. https://doi.org/10.1016/j.apsusc.2016.05.125.

(8) Mott, D.; Galkowski, J.; Wang, L.; Luo, J.; Zhong, C. J. Synthesis of Size-Controlled and Shaped Copper Nanoparticles. *Langmuir* **2007**, *23* (10), 5740–5745. https://doi.org/10.1021/la0635092.

(9) Deng, D.; Cheng, Y.; Jin, Y.; Qi, T.; Xiao, F. Antioxidative Effect of Lactic Acid-Stabilized Copper Nanoparticles Prepared in Aqueous Solution. *J. Mater. Chem.* **2012**, *22* (45), 23989–23995. https://doi.org/10.1039/c2jm35041f.

(10) Deng, D.; Jin, Y.; Cheng, Y.; Qi, T.; Xiao, F. Copper Nanoparticles: Aqueous Phase Synthesis and Conductive Films Fabrication at Low Sintering Temperature. *ACS Appl. Mater. Interfaces* **2013**, *5* (9), 3839–3846. https://doi.org/10.1021/am400480k.

(11) Begletsova, N.; Selifonova, E.; Chumakov, A.; Al-Alwani, A.; Zakharevich, A.; Chernova, R.; Glukhovskoy, E. Chemical Synthesis of Copper Nanoparticles in Aqueous Solutions in the Presence of Anionic Surfactant Sodium Dodecyl Sulfate. *Colloids Surfaces A Physicochem. Eng. Asp.* **2018**, *552*, 75–80. https://doi.org/10.1016/j.colsurfa.2018.05.023.

(12) Cure, J.; Glaria, A.; Collière, V.; Fazzini, P. F.; Mlayah, A.; Chaudret, B.; Fau, P. Remarkable Decrease in the Oxidation Rate of Cu Nanocrystals Controlled by Alkylamine Ligands. *J. Phys. Chem. C* **2017**, *121* (9), 5253–5260. https://doi.org/10.1021/acs.jpcc.6b12877.

(13) Ravi Kumar, D. V.; Kim, I.; Zhong, Z.; Kim, K.; Lee, D.; Moon, J. Cu(Ii)-Alkyl Amine Complex Mediated Hydrothermal Synthesis of Cu Nanowires: Exploring the Dual Role of Alkyl Amines. *Phys. Chem. Chem. Phys.* **2014**, *16* (40), 22107–22115. https://doi.org/10.1039/c4cp03880k.

(14) Zhang, T.; Hsieh, W. Y.; Daneshvar, F.; Liu, C.; Rwei, S. P.; Sue, H. J. Copper(i)-Alkylamine Mediated Synthesis of Copper Nanowires. *Nanoscale* **2020**, *12* (33), 17437–17449. https://doi.org/10.1039/d0nr04778c.

(15) Kanzaki, M.; Kawaguchi, Y.; Kawasaki, H. Fabrication of Conductive Copper Films on Flexible Polymer Substrates by Low-Temperature Sintering of Composite Cu Ink in Air. *ACS Appl. Mater. Interfaces* **2017**, *9* (24), 20852–20858. https://doi.org/10.1021/acsami.7b04641.

(16) Kamikoriyama, Y.; Imamura, H.; Muramatsu, A.; Kanie, K. Ambient Aqueous-Phase Synthesis of Copper Nanoparticles and Nanopastes with Low-Temperature Sintering and Ultra-High Bonding Abilities. *Sci. Rep.* **2019**, *9* (1), 1–10. https://doi.org/10.1038/s41598-018-38422-5.

(17) Lisiecki, I.; Pileni, M. P. Synthesis of Copper Metallic Clusters Using Reverse Micelles as Microreactors. *J. Am. Chem. Soc.* **1993**, *115* (10), 3887–3896. https://doi.org/10.1021/ja00063a006.

(18) Kim, N. S.; Hwang, S. Y.; Kim, E. Y.; Han, K. N. Synthesis of Copper Nano-Ink in Alcohol Media. *Jpn. J. Appl. Phys.* **2010**, *49* (5 PART 2), 5–9. https://doi.org/10.1143/JJAP.49.05EA04.

(19) Ankireddy, K.; Druffel, T.; Vunnam, S.; Filipič, G.; Dharmadasa, R.; Amos, D. A. Seed Mediated Copper Nanoparticle Synthesis for Fabricating Oxidation Free Interdigitated Electrodes Using Intense Pulse Light Sintering for Flexible Printed Chemical Sensors. *J. Mater. Chem. C* **2017**, *5* (42), 11128–11137. https://doi.org/10.1039/c7tc03522e.

(20) Xiong, J.; Wang, Y.; Xue, Q.; Wu, X. Synthesis of Highly Stable Dispersions of Nanosized Copper Particles Using L-Ascorbic Acid. *Green Chem.* **2011**, *13* (4), 900–904. https://doi.org/10.1039/c0gc00772b.

(21) Dabera, G. Di. M. R.; Walker, M.; Sanchez, A. M.; Pereira, H. J.; Beanland, R.; Hatton, R. A. Retarding Oxidation of Copper Nanoparticles without Electrical Isolation and the Size Dependence of Work Function. *Nat. Commun.* **2017**, *8* (1). https://doi.org/10.1038/s41467-017-01735-6.

(22) Kim In-Young, Joung Jae-Woo, S. Y.-A. *Reducing Agent for Low Temperature Reducing and Sintering of Copper Nanoparticles, and Method for Low Temperature Sintering Using the Same*; 2010.

(23) Cuya Huaman, J. L.; Sato, K.; Kurita, S.; Matsumoto, T.; Jeyadevan, B. Copper Nanoparticles Synthesized by Hydroxyl Ion Assisted Alcohol Reduction for Conducting Ink. *J. Mater. Chem.* **2011**, *21* (20), 7062–7069. https://doi.org/10.1039/c0jm04470a.
